# Bacteria Associated to Plants Naturally Selected in a Historical PCB Polluted Soil Show Potential to Sustain Natural Attenuation

**DOI:** 10.3389/fmicb.2017.01385

**Published:** 2017-07-25

**Authors:** Lorenzo Vergani, Francesca Mapelli, Ramona Marasco, Elena Crotti, Marco Fusi, Antonio Di Guardo, Stefano Armiraglio, Daniele Daffonchio, Sara Borin

**Affiliations:** ^1^Department of Food, Environmental and Nutritional Sciences, University of Milan Milan, Italy; ^2^Biological and Environmental Sciences and Engineering Division, King Abdullah University of Science and Technology Thuwal, Saudi Arabia; ^3^Department of Science and High Technology, University of Insubria Como, Italy; ^4^Municipality of Brescia, Museum of Natural Sciences Brescia, Italy

**Keywords:** soil pollution, natural attenuation, bacterial diversity, rhizosphere, polychlorobiphenyls, plant growth promotion, *bphA* gene

## Abstract

The exploitation of the association between plants and microorganisms is a promising approach able to boost natural attenuation processes for soil clean-up in vast polluted areas characterized by mixed chemical contamination. We aimed to explore the selection of root-associated bacterial communities driven by different plant species spontaneously established in abandoned agricultural soils within a historical polluted site in north Italy. The site is highly contaminated by chlorinated persistent organic pollutants, mainly constituted by polychlorobiphenyls (PCBs), together with heavy metals and metalloids, in variable concentrations and uneven distribution. The overall structure of the non-vegetated and root-associated soil fractions bacterial communities was described by high-throughput sequencing of the 16S rRNA gene, and a collection of 165 rhizobacterial isolates able to use biphenyl as unique carbon source was assayed for plant growth promotion (PGP) traits and bioremediation potential. The results showed that the recruitment of specific bacterial communities in the root-associated soil fractions was driven by both soil fractions and plant species, explaining 21 and 18% of the total bacterial microbiome variation, respectively. PCR-based detection in the soil metagenome of bacterial *bphA* gene, encoding for the biphenyl dioxygenase α subunit, indicated that the soil in the site possesses metabolic traits linked to PCB degradation. Biphenyl-utilizing bacteria isolated from the rhizosphere of the three different plant species showed low phylogenetic diversity and well represented functional traits, in terms of PGP and bioremediation potential. On average, 72% of the strains harbored the *bphA* gene and/or displayed catechol 2,3-dioxygenase activity, involved in aromatic ring cleavage. PGP traits, including 1-aminocyclopropane-1-carboxylic acid deaminase activity potentially associated to plant stress tolerance induction, were widely distributed among the isolates according to *in vitro* assays. PGP tested *in vivo* on tomato plants using eleven selected bacterial isolates, confirmed the promotion and protection potential of the rhizosphere bacteria. Different spontaneous plant species naturally selected in a historical chronically polluted site showed to determine the enrichment of peculiar bacterial communities in the soil fractions associated to the roots. All the rhizosphere communities, nevertheless, hosted bacteria with degradation/detoxification and PGP potential, putatively sustaining the natural attenuation process.

## Introduction

Polychlorinated biphenyls (PCBs) are highly stable, hydrophobic and persistent organic pollutants. Due to their lipophilic nature, they bioaccumulate and biomagnify through the food web and can have a broad range of toxic effects on humans (Turrio-Baldassarri et al., 2009; Letcher et al., 2010; Quinete et al., 2014; IARC, 2015). Physico-chemical remediation techniques of PCB polluted soils are not sustainable environmentally and economically for extended contaminations and the properties of these molecules make them recalcitrant to biodegradation, impairing the efficacy of bioremediation technologies. However, PCB pollution is a worldwide problem associated to their past production and utilization in industrial facilities (Passatore et al., 2014). PCBs strongly bind to the soil organic matter, resulting into low bioavailability both for plant uptake and microorganism metabolism (Sinkkonen and Paasivirta, 2000). Nonetheless, several studies showed that PCB-polluted soils host bacterial communities endowed with aerobic degradation abilities and harboring the biphenyl dioxygenase (*bph*) operon, the most studied and widespread pathway sustaining the aerobic biodegradation ability (Abraham et al., 2002; Leigh et al., 2006; Leigh et al., 2007; Kurzawova et al., 2012). Plants establish mutual beneficial interactions with selected bacterial populations, which promote nutrient uptake and enhance stress tolerance against pollutants, such as PCBs, in turn decreasing their phytotoxicity (Ma et al., 2011; Abhilash et al., 2016). Root exudates, besides creating a selective ecological niche for specific bacteria, may contain molecules that can induce the expression of genes involved in the PCB degradation pathways, such as the *bph* operon, in turn stimulating PCB degradation in the rhizosphere (Toussaint et al., 2012; Pham et al., 2015). Rhizoremediation, the exploitation of such positive interactions between plants and bacteria for reclamation of polluted soils, represents a sustainable alternative for clean-up of recalcitrant organic pollutants in extended areas (Vergani et al., 2017). Soil natural attenuation potential therefore relies on the presence, diversity and activity of the resident microbiota, driven by the selecting activity of autochthonous plants.

In this work, we studied the phylogenetic and functional diversity of the microbiota associated to the root-soil fractions of the three spontaneous plant species *Medicago sativa, Centaurea nigrescens*, and *Dactylis glomerata*, naturally established in a former agricultural field located within the heavily polluted SIN (National Priority Sites) site Caffaro in north Italy. This is a large area polluted by the activity of the former Caffaro chemical factory and includes more than 100 ha of former agricultural areas (Di Guardo et al., 2017). It presents a mixed and uneven contamination of chlorinated persistent organic pollutants, mainly PCBs, heavy metals and metalloids and several other contaminants including dioxins, furans, tetra-chloromethane (Turrio-Baldassarri et al., 2007; Di Guardo et al., 2017). PCBs were produced by Caffaro for 46 years and banned in Italy in 1984. To avoid pollutant exposure to humans, economic activities, including agriculture, have been banned in 2002 in the SIN Caffaro area. In the last 12 years only natural attenuation processes occurred, with the establishment of spontaneous plants resisting phytotoxic effects. Remediation strategies for such a large and complex site represent a challenge raising the interest for the soil self-depuration potential, which could be exploited and boosted by phyto-rhizoremediation approaches. A basic question is whether PCB degradation potential is present in the bacterial communities enriched by plants able to grow in the polluted soil, mediated by the selection force determined by the rhizosphere effect. We compared the bacterial communities in the root-associated soil fractions with that of the non-vegetated soil, through 16S rRNA gene phylogenomics and the amplification of the *bphA* gene, encoding for the α sub-unit of the biphenyl dioxygenase enzyme, a proxy for the PCB degradation ability (Iwai et al., 2010; Sylvestre, 2013; Vergani et al., 2017). By looking to the culturable bacterial fraction, we assessed whether bacteria represent a potentially exploitable resource for rhizoremediation purposes, by assessing their PGP capacity and PCB biodegradation potential.

## Materials and Methods

### Site Description, Plants and Soil Sampling

Non-vegetated and root-associated soil fractions were collected in the SIN Caffaro, a site located in Northern Italy (Brescia municipality), within the sampling area A, a former agricultural grassland field that was previously chemically characterized and contained PCBs and other chlorinated pollutants in concentrations often exceeding the safety limits (Di Guardo et al., 2017). The root system of three spontaneous plant species was collected from triplicate specimens for each plant species. Plants were identified basing on their morphological traits as *M. sativa* L. (MS), *C. nigrescens* Willd. (CN), and *D. glomerata* L. (DG). These plant species were among the most widely widespread in the meadows present in the agricultural areas of the SIN Caffaro, according to a recent survey (Armiraglio et al., 2009). Non-vegetated bulk soil (B) was collected in triplicate in the same sampling station. Samples were transferred to the laboratories and the different soil fractions (as defined below) were separated within 4 h from collection as described in Marasco et al. (2012). Soil fractions were stored at -20°C for molecular analyses and at 4°C for bacterial isolation.

### DNA Extraction and Analyses of the Bacterial Community Structure

Total DNA extraction was performed from 0.25 g of each of the three non-vegetated bulk soil (B), root surrounding soil (S) and rhizosphere soil (R) replicates, using the PowerSoil DNA kit (MoBio) according to the manufacturer’s protocol. Illumina tag screening of the V3–V4 hypervariable regions of the 16S rRNA gene was applied on DNA of R, S, and B triplicate samples using the primers 341F and 785R (Klindworth et al., 2013). The obtained sequences were analyzed using a combination of the UPARSE v8 (Edgar, 2013) and the QIIME v1.8 (Caporaso et al., 2010b) softwares. Briefly, raw forward and reverse reads for each sample were assembled into paired-end reads considering a minimum overlapping of 50 nucleotides and a maximum of one mismatch within the region using the fastq-join algorithm^1^. The paired reads were then quality filtered, the primer sequences were removed and the individual sample files were merged in a single fasta file. This file was imported in UPARSE where operational taxonomic units (OTUs) of 97% sequence similarity were formed and chimeras were removed using both *de novo* and reference-based detection. For reference chimera detection, the “Gold” database containing the chimera-checked reference database in the Broad Microbiome Utilities^2^ was used. Taxonomy was assigned to the representative sequences of the OTUs in QIIME using UClust (Edgar, 2010) and searching against the latest version of the Greengenes database (McDonald et al., 2012). After the removal of reads affiliated to chloroplast, Archaea and unassigned sequences, a total of 1319653 high-quality merged paired-end reads with an average length of 450 bp were obtained. All the samples analyzed presented Good’s coverage values ranging from 90 to 99 capturing sufficient diversity with an adequate sequencing depth (Supplementary Table 1).

The OTU table, composed by 3587 OTUs, and the phylogenetic tree were calculated with FastTree2 (Price et al., 2010) using default parameters and the PyNast-aligned (Caporaso et al., 2010a) representative sequences as an input. The OTU table and the phylogenetic tree were used as inputs for the subsequent analyses of alpha- and beta-diversity. Bray–Curtis distance matrix on the log transformed OTU table was used to perform a Principal Coordinates Analysis, Canonical Analysis of Principal coordinates (CAP) and to conduct a permutational multivariate analyses of variance (PERMANOVA). Statistical analyses were conducted in PRIMER v. 6.1, PERMANOVA++ for PRIMER routines (Anderson et al., 2008) to test differences in bacterial community composition among the three soil fractions and among the three plant species. A first analysis was conducted in a one-way anova to explore difference among the fraction considering as a factor “Fraction” as fixed and orthogonal (three levels: rhizosphere/root surrounding soil/non-vegetated bulk). A second analysis was conducted exploring difference of the microbial assemblage among plants and fractions using the factor “Plant” (three levels: *M. sativa, C. nigrescens* and *D. glomerata*) and the factor “Fraction” (two levels: rhizosphere and root surrounding soil) both as a fixed and orthogonal, and their interaction (Fraction × Plant). Prior to perform the statistical analysis we verify that the data were not over-dispersed using PERMDISP for the factor “Fraction” (*F*_1,18_ = 3.61, *p* = 0.21) and the factor “Plant” (*F*_2,15_ = 9.36, *p* = 0.06). The shared OTUs among different fractions and plant species have been defined by Venn-diagram analysis using the software available at http://bioinformatics.psb.ugent.be. Diversity indexes were calculated using PAST (Hammer et al., 2001) and their statistical difference was evaluated with the analysis of variance considering the index as response variable and “Fraction” and “Plant” as explanatory categorical variable (see above for details). Raw sequences have been deposited at the ENA European Read Archive under accession number from SAMN07167786 to SAMN07167806, BioProject PRJNA388028, Submission ID SUB2728610.

16S rRNA gene quantitative PCR (qPCR) was performed in a reaction volume of 15 μl with 1 μl of template using universal primers 27F and 1492R and the SsoAdvanced^TM^ Universal SYBR^®^ Green Supermix (Bio-Rad). Thermal protocol was set up as follow: 98°C (3 min), then 40 cycles at 98°C (15 s), 58°C (30 s) and 72°C (1 min). Starting DNA concentration (ng/μl) was measured using a PowerWave HT Microplate Spectrophotometer (BioTek) and the number of 16S rRNA copies obtained with qPCR was normalized with the DNA concentration.

### Bacteria Isolation and Identification

For bacterial isolation, the R samples obtained from the triplicate plants of the same species were pooled and homogenized. One gram of the resulting soil was suspended in 9 ml of physiological solution (0.9% NaCl), diluted in 10-fold series and plated onto mineral medium (Uhlik et al., 2011), adding biphenyl crystals on the plate lid as unique carbon source. Colonies were randomly picked after 1 week of incubation at 30°C, after the appearance of a stable number of colonies, and were spread three times on the same medium to obtain pure bacterial cultures. A collection of 165 biphenyl-utilizing rhizobacterial strains was established (52 isolates from MS, 56 from DG and 57 from CN) and cryopreserved in 25% glycerol at -80°C. Strain code includes a different number according to the plant of origin (1: MS, 2: CN, 3: DG).

The genomic DNA of each isolate was extracted through boiling cell lysis (Ferjani et al., 2015). Bacterial strains were identified through 16S rRNA gene amplification and partial sequencing (Macrogen, Rep. of South Korea) as described by Mapelli et al. (2013). 16S rRNA nucleotide sequences were subjected to BLAST search (using the blastn suite) and were deposited in the ENA database under accession numbers LT838007–LT838169.

### *bphA* Gene Detection, Quantification and Sequencing in Soil Metagenomes and Strain Genomes

The presence of the genes encoding for biphenyl dioxygenase α subunit (*bphA*) was assessed in the metagenome of soil samples (B, S, and R) through PCR as described by Iwai et al. (2010), using the primers BPHD-F3/R1, and further confirmed using 512F and 674R primer set (Leewis et al., 2016b). The latter primer set was also used for a *bphA* gene qPCR assay as described by Leewis et al. (2016b) and the relative abundance of *bphA* gene copies was expressed as a ratio over the total community 16S rRNA gene copy number. Bacterial isolates were grown overnight in Tryptic Soy Broth medium and then subjected to CTAB – phenol chloroform DNA extraction (Chouaia et al., 2010). *BphA* gene amplification was performed with 2 μl of DNA template in a final volume of 30 μl with primers 463F/674R (Petric et al., 2011), at the following conditions: buffer 1X, MgCl_2_ 1.8 mM, dNTPs 0.2 mM, primers 1 μM, Taq 1.5 U per reaction. PCR thermal protocol was set up as follows: 10 min at 95°C, then 40 cycles of 95°C (15 s), 65°C (1 min), 72°C (2 min) and a final elongation step of 10 min at 72°C. All PCR reactions were performed utilizing FastStart^TM^ High Fidelity PCR System (Roche). Genomic DNA of the model PCB-degrading strain *Paraburkholderia xenovorans* LB400 (DSMZ, Germany), was used as positive control for all *bphA* PCR reactions. PCR results were visualized on 1.2% agarose gel and PCR products that did not show the presence of aspecific bands were sequenced at Eurofins Genomics (Italy). Sequences were then identified using the BLASTn suite of the NCBI website^3^ and a Neighbor-Joining phylogenetic tree was then built using MEGA 5.1 (Tamura et al., 2011), computing the evolutionary distances using the Jukes–Cantor method. Nucleotide sequences were deposited in the ENA database under accession numbers LT840193– LT840239.

### *In Vitro* Characterization of Bacterial Isolates for PGP Traits and Bioremediation Potential

*In vitro* screening for the presence of activities related to plant growth promotion (PGP) was performed for the entire bacteria collection. Inorganic phosphate solubilization and the production of indole-3-acetic acid (IAA), ammonia, protease and exopolysaccharides (EPS) were assessed as described by Cherif et al. (2015); 1-aminocyclopropane-1-carboxylic acid (ACC) deaminase activity was evaluated according to Belimov et al. (2005). Strains were also tested for abiotic stress resistance, namely the ability to grow at 4 and 42°C, and in presence of 5% NaCl and 20% polyethylene glycol (PEG) supplemented to the medium (Cherif et al., 2015). Catechol 2,3-dioxygenase activity was tested following the protocol described by Margesin et al. (2003).

### *In Vivo* Assessment of Plant Growth Promotion of Tomato

Tomato seeds were sown in commercial non-sterile soil placed in trays and, after 1 week, uniform-sized seedlings were selected, each transplanted in separated 0.3 kg commercial soil pots and maintained under greenhouse conditions (≈110 photons m^-2^ s of light for 12 h during the day and average temperature of 25°C). A subset of 11 rhizobacteria strains, selected based on a cluster analysis performed using MVSP (Kovach, 1999) combining the PGP activities and the abiotic stress tolerance traits, were inoculated separately on tomato plants. One week after transplantation, the tomato plants (*n* = 5 for each treatment) were fertilized once with a bacterial suspension of the selected strains at a final concentration of 10^8^ cells g^-1^ of soil (fresh weight). The inocula have been prepared according to Rolli et al. (2015) resuspending the bacterial cells in sterile tap water. Non-inoculated plants (*n* = 5) were irrigated with the same amounts of sterile tap water and used as control. After 30 days, plants were harvested for the measurement of shoot/root length and weight. Statistical analysis was performed by a pairwise comparison between each bacterial strain inoculated with the negative control, using Student’s *t*-test.

## Results

### Diversity and Degradation Potential of Bacterial Communities Associated to Non-vegetated and Root-Associated Soil Fractions in the SIN Caffaro

**FIGURE 1:**
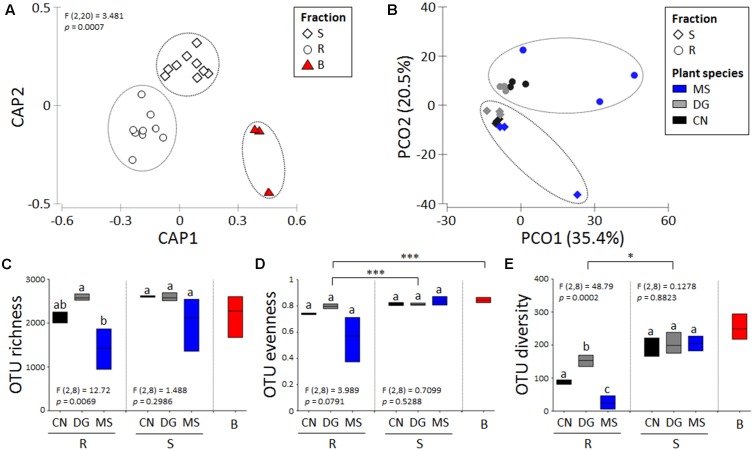
Analysis of bacterial community’s structure and diversity of soil samples associated to spontaneous plants at SIN Caffaro. **(A)** Constraint (CAP) and **(B)** Unconstraint analysis of principal coordinates of the OTU_97_ obtained from 16S rRNA gene sequencing of the bacterial communities considering the different soil fractions **(A)** and the root-associated soil fractions according to the plant species **(B)**. **(C)** OTU_97_ Richness, **(D)** OTU_97_ Evenness and **(E)** OTU_97_ diversity. Statistical analysis are indicated in within the plant species comparing each fraction while asterisks indicate the statistical significance among fractions (for details see the text). The letters R, S, and B indicate, respectively, the rhizosphere, root surrounding soil and non-vegetated bulk soil.

The β-diversity of bacterial communities colonizing non-vegetated bulk soil (B) and root-associated soil fractions (rhizosphere, R, and root surrounding soil, S) in the SIN Caffaro was significantly different (**Figure 1A**, PERMANOVA, *F*_2,18_:3.48, *p* = 0.0007, Supplementary Table 2A). Considering only the R and S soil fractions associated to the three plant species *M. sativa* (MS)*, C. nigrescens* (CN) and *D. glomerata* (DG), the bacterial population assemblage was significantly driven by both plant species and soil fraction (**Figure 1B**, PERMANOVA, *F*_1,12_ = 4.72, *p* < 0.001 and PERMANOVA, *F*_2,12_ = 3.21, *p* < 0.05, respectively, Supplementary Table 2B) but not by their interaction (PERMANOVA, *F*_2,17_ = 1.67, *p* > 0.05, Supplementary Table 2B). Quantification of the individual factors’ contributions to the observed bacterial community variations, determined by PERMANOVA of Bray–Curtis, showed that the two factors “Fraction” and “Plant species” equally contributed to determine the observed bacterial microbiome variation, explaining 21 and 18% of the variation, respectively (Supplementary Table 3), followed by the interaction of these two factors (11%). For the three fractions, not significant differences have been retrieved considering the OTU richness, but among the rhizosphere the MS and CN plants presented a lower number of OTUs (**Figure 1C**). OTU evenness was instead significantly different among fractions (ANOVA, *F*_2,20_ = 21.63, *p* < 0.001) but not between plants (**Figure 1D**). OTU diversity, calculated as inverse Simpson diversity index, was significantly lower in R compared to B and S (ANOVA, *F*_2,20_ = 4.7, *p* < 0.05). While not significant differences have been observed among the different plants in S fraction, plant species determined a strong effect in R fraction where DG hosted the significantly highest diversity, followed by CN and MS (**Figure 1E**).

**FIGURE 2:**
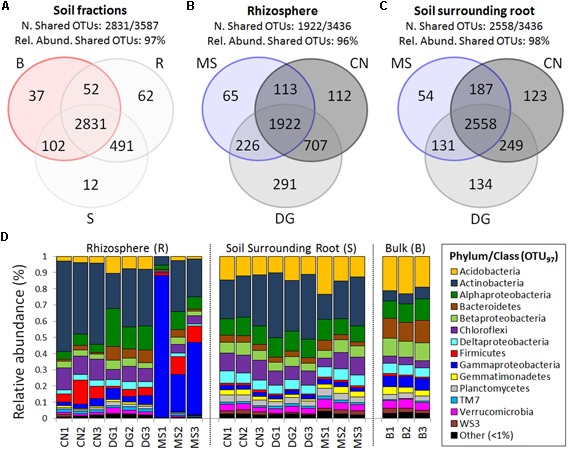
Shared microbiome and taxonomic diversity of bacterial communities associated to non-vegetated bulk soil and root-associated soil fractions of plants grown in the SIN Caffaro. Venn diagrams showing the shared and exclusive bacterial OTUs of **(A)** the three soil fractions (R, S, and B). Specific and shared OTUs among the three plants in rhizosphere soil **(B)** and soil surrounding root **(C)** have been reported. Number of the shared OTUs and their relative abundance in all soil fractions, rhizosphere and soil surrounding root have been listed on the top of each Venn diagram. **(D)** Relative abundance of different bacterial phylum/class in the non-vegetated bulk soil (B), rhizosphere (R), and soil surrounding root (S) of the three plants (MS, CN, and DG) representing OTUs showing more than 0.001% relative abundance of all reads. Phyla/classes representing less than 1% out of total reads were grouped as ‘Other.’

The three soil fractions collected in the SIN Caffaro showed (i) low number of specific OTUs typical of each fraction, and (ii) a consistent group of shared OTUs (2831/3587) accounting the 97% of the 16S rRNA sequences (**Figure 2A** and Supplementary Table 4A). The rhizosphere and root surrounding soil associated to the three plant species shared a total of 1922/3436 and 2558/3436 OTUs, respectively, accounting up to 98% of relative abundance in S (**Figures 2B,C** and Supplementary Tables 4B,C). The high number of shared OTUs in non-vegetated and plant-associated soils indicated that all the fractions hosted bacterial communities with similar phylogenetic composition and different in the structure, as demonstrated by the analysis of Beta and Alpha-diversity (**Figure 1**).

The taxonomic affiliation of OTUs (**Figure 2D** and Supplementary Tables 5, 6) revealed that the soil fractions hosted 37 bacterial phyla, 105 classes (99.3% sequences classified), 153 orders (89% classified), 167 families (67% sequences classified) and 162 genera (23% sequences classified). The most represented phyla were present in all the soil fractions (R, S, and B): *Proteobacteria* 34% (among these *Gammaproteobacteria* 16% and *Alphaproteobacteria* 10%), *Actinobacteria* 30%, *Chloroflexi* (8%), and *Acidobacteria* (8%). The three soil fractions were nevertheless dominated by different phyla/classes. In the non-vegetated soils *Proteobacteria* (on average 35%, mainly represented by *Alphaproteobacteria* and *Betaproteobacteria*) was the prevalent phylum followed by *Acidobacteria* (22%) and *Bacteroidetes* (12%) (**Figure 2D**). *Proteobacteria* dominated also the rhizospheres with *Gammaproteobacteria* (26%) and *Alphaproteobacteria* (9%) as the main *Proteobacteria*-classes, while in S fraction the *Actinobacteria* was the dominant phylum (30%).

Moving from the root-associated fractions (R and S) to the non-vegetated bulk soil, a strong reduction of the *Actinobacteria* phylum was observed in the bacterial communities, from an average of 33% in R to an average of 6% in B (Supplementary Table 5). An opposite trend has been observed for *Acidobacteria*, *Bacteroidetes, Verrucomicrobia*, and *Gemmatimonadetes* phyla that were enriched in the non-vegetated bulk soil and strongly limited in R and S fractions (Supplementary Table 5). Finally, while *Firmicutes* bacteria were enriched in R (7%) respect to the other two fractions (1%), *Chloroflexi* were more prevalent in S (10%) respect to R (7%) and B (4%). Difference in taxa enrichment and selection were observed also according to the plant species in both R and S fractions (Supplementary Table 7). The MS and CN bacterial communities in R fractions were dominated by *Gammaproteobacteria* (52%) and *Actinobacteria* (50%) respectively, determining a higher dominance index value (0.32 and 0.28) in these communities respect to the DG one (0.15), while DG hosted a more equally distributed bacterial community with Evenness index of 0.62. An opposite trend has been observed in the S fraction, where DG was dominated by *Actinobacteria* (37%) while MS and CN presented several taxa equally distributed within the bacterial communities (Evenness 0.72 and 0.69, respectively, Supplementary Table 7).

The *bphA* gene was detected, by means of qualitative PCR using different primer sets, in the metagenomes of all B, S and R samples. Despite that, the quantification of *bphA* gene with primers 512F and 674R was possible only for two replica of the CN rhizosphere (*bph*A/16S rRNA ratio, CN R2: 0.56 ± 0.03; CN R3: 0.50 ± 0.08) and one replica of the DG S (*bph*A/16S rRNA ratio, DG S2: 0.08 ± 0.004), showing the highest *bphA*/16S rRNA gene ratio in the CN rhizosphere.

### Identification of Rhizosphere Biphenyl-Utilizing Bacterial Isolates

One-hundred sixty-five bacterial strains have been isolated from the rhizosphere of the three plant species MS, CN, DG in mineral medium supplemented by biphenyl as unique carbon source, hence considered as potential biphenyl degraders. The strains belonged only to 3 phyla, *Actinobacteria* (75, 51, and 52% in MS, CN, and DG collections, respectively), *Proteobacteria* (17, 30, and 32% in MS, CN, and DG collections, respectively) and *Bacilli* (8, 11, and 13%, in MS, CN, and DG collections, respectively). All the isolates belonged to phyla that were present in all the metagenome 16S rRNA high-throughput sequencing libraries, even though with a different relative abundance. All the isolates exhibited sequence identity with the closest described species in NCBI database higher than 97% (Supplementary Table 8) and their phylogenetic affiliation at the genus level is represented in Supplementary Figure 1. Among *Gammaproteobacteria, Pseudomonas* species were widespread in the three sub-collections, and constituted 15, 23 and 13% of MS, CN, DG rhizosphere, respectively. *Acinetobacter* was isolated only from CN and DG rhizospheres (5 and 16%, respectively). Among the phylum *Bacilli*, the genus *Bacillus* was the most represented, isolated from the rhizosphere of all plant species (7, 13, and 6% in CN, DG, and MS, respectively). Seventy-three percent of all the isolates from the rhizosphere of the three plant species belonged to three genera: *Arthrobacter* (18, 14, 10 strains from DG, MS, CN rhizospheres, respectively), *Microbacterium* (7, 24, 11 strains from DG, MS, CN rhizospheres, respectively), *Pseudomonas* (7, 8, 13 strains from DG, MS, CN rhizospheres, respectively). MS rhizosphere showed the lowest number (6) of cultivable genera within the biphenyl-utilizing bacteria, widely dominated by *Microbacterium* (44% of the isolates) and *Arthrobacter* (29% of the isolates), compared with CN and DG rhizosphere that included 11 genera.

### Functional Characterization of the Cultivable Rhizosphere Biphenyl-Utilizing Bacteria

**FIGURE 3:**
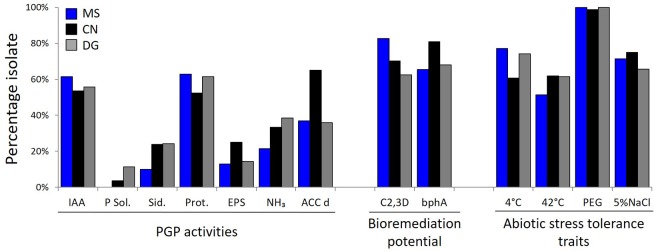
Characterization of the bacteria collection for plant growth promotion (PGP) traits and degradation capacity. Percentage of strains isolated from MS (blue), CN (black), DG (gray) rhizosphere that resulted positive to PGP and degradation screenings. Among PGP activities: IAA indolacetic acid production, P. Sol. = inorganic phosphate solubilisation, Sid. = siderophore production, Prot. = protease production; EPS = exopolysaccharides release, NH_3_ = ammonia production, ACCd = ACC-deaminase activity. Activities related with bioremediation potential: C 2,3 D = 2,3-catechol dioxygenase activity and bphA = positive PCR amplification of the *bphA* gene. Among abiotic stress tolerance: 4°C exposure, 42°C exposure, 20% PEG (to simulate osmotic stress) and salinity tolerance with a 5% NaCl exposure.

The isolate collection has been screened *in vitro* for PGP activities, bioremediation potential and abiotic stress resistance (results are detailed for each isolate in Supplementary Table 8). Production of IAA and proteases were the PGP related activities most represented among the collection, present in more than 50% of the strains, in similar percentages among sub-collections isolated from each plant species (**Figure 3**). A large fraction of the collection produced ammonia (21, 33, and 39% of the MS, CN, and DG strains, respectively), EPS (13, 25, and 14%, respectively) and siderophores (10, 24, and 24%, respectively). ACC deaminase activity was detected in higher percentage among CN (65%) than MS (37%) and DG (36%) sub-collections. Phosphate solubilization activity was present in a small proportion among CN (4%) and DG (11%) isolates, while it was absent in MS sub-collection. Concerning the biodegradation potential, catechol 2,3-dioxygenase activity was widespread in the entire collection with 83, 70, and 63% of the MS, CN, and DG active isolates, respectively. Biphenyl dioxygenase α subunit gene (*bphA*) was detected in the genomic DNA of 65, 81, and 68% of the MS, CN, and DG isolates, respectively. PCR products presenting one single band of the expected size (211 bp) in agarose gel electrophoresis were sequenced, obtaining 45 partial *bphA* gene sequences (9, 19, and 17 from MS, CN, and DG isolates, respectively). Eighty percent of the sequences showed high nucleotide identity (>99%) with *Pseudomonas pseudoalcaligenes* KF707 *bphA*, and the remaining ones showed >99% nucleotide identity with *Rhodococcus wratislaviensis* strain P13 *bphA1* and *R. opacus bphA1* genes (Supplementary Table 9). The *bphA* sequences were clustered in a phylogenetic tree with *bphA* sequences of reference strains having demonstrated PCB degradation ability. The sequences were clustered in two groups, both including known PCB-degraders (Supplementary Figure 2): *Pseudomonas* KF707-like sequences clustered together with *Paraburkholderia xenovorans* LB400, while *Rhodococcus*-like sequences grouped separately.

All strains were able to cope with the osmotic stress induced by the addition of 20% of PEG in the growth medium (Supplementary Table 2). The majority of the strains (71% on average) from each plant species proved to grow in presence of salt in the growth medium, while a percentage comprised between 58 and 71% tolerated high (42°C) and low (4°C) temperatures for growth (**Figure 3** and Supplementary Table 8).

A total of 11 strains has been selected basing on PGP activities and abiotic stress tolerance traits (Supplementary Figure 3) and have been tested *in vivo* on tomato as a model plant. The strains were affiliated to different species belonging to the genera *Pseudomonas*, *Acinetobacter*, *Arthrobacter*, and *Curtobacterium*, and presented an array of different *in vitro* PGP traits, abiotic stress tolerance and bioremediation potential (Supplementary Table 10).

**FIGURE 4:**
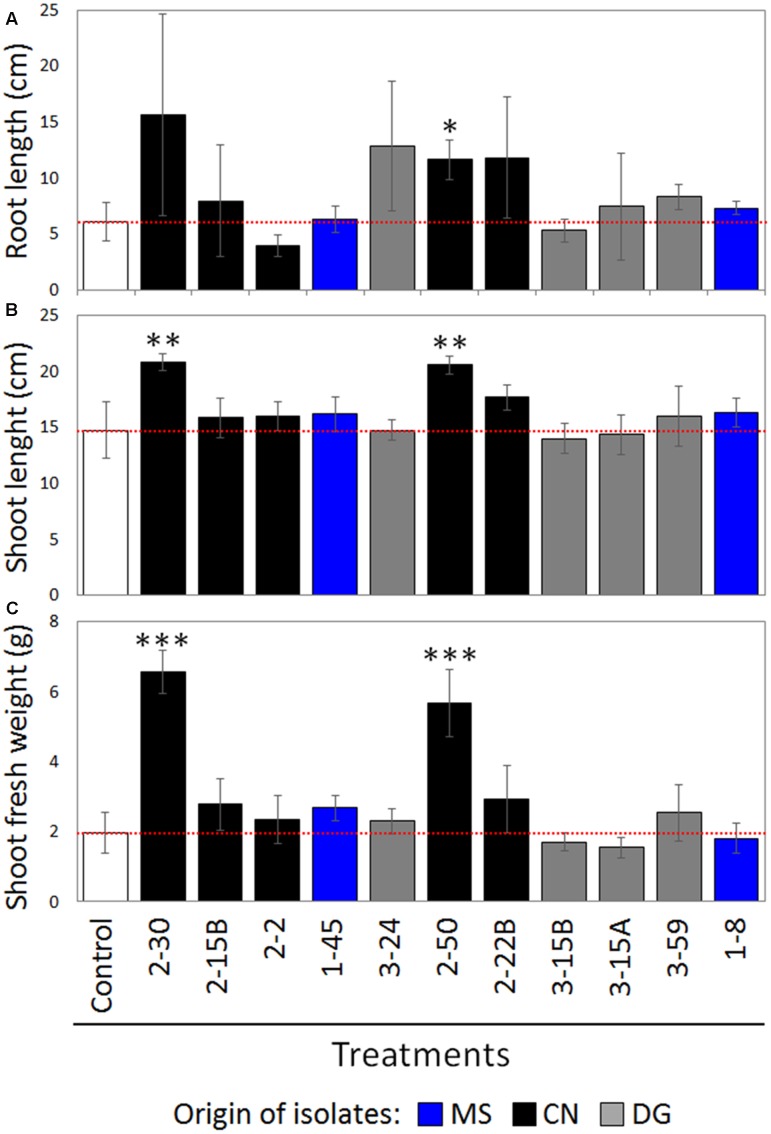
Results of *In vivo* plant growth promoting test on tomato under greenhouse conditions, according to **(A)** shoots length, **(B)** root length, and **(C)** fresh weight measurement. Screened strains and plant measurement results are reported on the *X*-and *Y*-axes, respectively. The data were calculated as average of five plants per treatment and Student’s *t*-test was adopted to statistically analyze the data. The star indicates statistically significant differences (^∗^*p* < 0.05, ^∗∗^0.001 < *p* < 0.05, ^∗∗∗^*p* < 0.001).

Despite showing only 1 and 2 *in vitro* PGP traits, respectively, strains 2–30 and 2–50, both isolated from CN plant and affiliated to different species of the genus *Arthrobacter* (Supplementary Table 10), significantly promoted plant growth compared to the non-inoculated control (**Figure 4**). Both strains mainly affected shoot development, significantly increasing shoot length (*p* < 0.01) and fresh weight (*p* < 0.001). While 2–30 did not showed effect on the root development, 2–50 significantly promote also the root length (*p* < 0.05). Strain 2–50 possesses, moreover, both the tested bioremediation potential traits (Supplementary Table 10).

## Discussion

### Plant Influence on Soil Bacterial Community Structure and Degradation Potential

In vegetated soils physico-chemical properties, together with plant root exudation and turnover, shape the structure of the microbial community. Resulting in the so-called “rhizosphere effect,” every plant species presents a specific pattern of root exudation and interacts with the soil community selecting a specific microbiota recruited from the surrounding soil (Haichar et al., 2008; Berg and Smalla, 2009). Here we compare the bacterial communities inhabiting the rhizosphere and root surrounding soils of three spontaneous plant species with a non-vegetated bulk soil from a site heavily contaminated with chlorinated persistent organic pollutants and heavy metals, demonstrating that both the soil fraction and the plant species cooperated in the selection of a specific bacterial assemblage in the root-associated soils. The bacterial communities inhabiting all soil fractions hosted the typical phyla that dominate soil ecosystems, i.e., *Acidobacteria, Actinobacteria, Bacteroidetes, Chloroflexi*, and *Proteobacteria* (Bulgarelli et al., 2013). R and S fractions, that are directly under plant root influence, showed an increase of *Actinobacteria* and *Proteobacteria.* Within the *Actinobacteria*, the order *Gaiellales* was retrieved at higher percentage in all the plant-associated fractions and in particular in the CN rhizosphere. This bacterial order was previously detected in the root system of rice (Hernández et al., 2015) and maize planted on heavy metals polluted soil (Touceda-Gonzalez et al., 2015), however, it is still poorly studied and includes only one described species (Albuquerque et al., 2011). Plant exudates enriched also the orders *Sphingomonadales* and *Pseudomonadales* in the DG and MS rhizospheres, respectively. Members of these orders have already been retrieved in PCB polluted soils, involved in biphenyl degradation and for some genera also in PCB degradation (Leigh et al., 2007; Hu et al., 2015; Jayanna and Gayathri, 2015; Leewis et al., 2016b). The phylum *Chloroflexi*, comprising a well known group of anaerobic PCB degraders (Jugder et al., 2015), was retrieved at relative abundance higher than 1% exclusively in the S fractions, being particularly enriched in MS1. It is possible that an anoxic microniche occurred in this sample, allowing the selection of *Chloroflexi* members, in agreement with previous finding of *Chloroflexi* enrichment in the rhizosphere of *M. sativa* during a PCB polluted soil phytoremediation trial (Tu et al., 2011) and of *Sparganium* sp. during biostimulation of the autochthonous microbiota in historically PCB polluted sediments (Di Gregorio et al., 2013).

Studies on a hydrocarbon-contaminated soil proved that the pollutant concentration rather than rhizosphere effect of planted willows had a major role in shaping the bacterial community (Bell et al., 2014; Yergeau et al., 2014). In a previous work (Di Guardo et al., 2017) we suggested that soil pollutant profiles of different former agricultural fields in the SIN Caffaro acted as drivers of the bacterial community composition. Here we demonstrated that in one of the previously analyzed fields, the rhizosphere effect of spontaneous plants adapted to the occurring soil contamination significantly influenced bacterial assemblages in the root-associated soil fraction, R and S. We speculate that in the rhizosphere an enhanced pollutant degradation, according to the higher abundance of taxa previously associated to PCB metabolism, contributed to shape bacterial diversity (Leewis et al., 2016a; Ridl et al., 2016). Our results indicated that autochthonous vegetation, not specifically selected for rhizoremediation efficiency, but rather naturally adapted to counteract phytotoxicity and the specific polluted soil conditions, can establish a strong relationship with the soil bacterial community, potentially sustaining PCB detoxification and in turn the natural attenuation process. The diversity of R bacterial communities was significantly influenced by the three plant species, which selected a root-associated microbiome having a peculiar structure. Despite this, the occurrence of a high percentage of OTUs shared among the different soil fractions, may suggest a strong selection force toward specific taxa constituted by the high pollution level, and possibly related to contaminant detoxification.

Different plant species, including MS, were previously demonstrated to enrich and stimulate the PCB-degrading bacterial communities, inducing an increase of *bphA* gene copy number and its expression levels in the rhizosphere compared to the non-vegetated bulk soil (Tu et al., 2011; Li et al., 2013; Pagé et al., 2015). In this work, the *bphA* gene was detected in the metagenome of all the soil fractions and could be quantified in CN rhizosphere and DG soil surrounding roots. The copy number ratio between this gene and the 16S rRNA bacterial gene was higher in R than in S samples, leading to speculate that bacterial populations harboring degrading potential were enriched in the rhizosphere fraction. However, the spread of *bphA* gene in the bacterial microbiota of the SIN Caffaro soil indicated an intrinsic PCB attenuation potential in this site, regardless of soil fraction or plant species, suggesting a stronger role of edaphic conditions rather than vegetation.

### Isolates Potential for Rhizoremediation

The root system of plant growing on other PCB polluted sites, was shown to host PCB metabolizing bacteria (Leigh et al., 2006; Ionescu et al., 2009). Hence, we applied a cultivation approach to identify indigenous bacteria in the rhizosphere of plants naturally able to cope with the high level of pollution in the SIN Caffaro, having PGP and PCB degrading potentials. The synergistic effect of these traits has indeed the potential to sustain rhizoremediation approaches, in which both plants and bacteria are involved in remediation (Vergani et al., 2017). The cultivation approach applied to MS, CN, DG rhizospheres, due to the selective conditions determined by biphenyl as unique carbon source, led to isolate only bacteria species belonging to three of the thirty-seven phyla identified in the soil metagenome by 16S rRNA high-throughput sequencing (*Actinobacteria, Proteobacteria*, and *Firmicutes*). The high taxonomic similarity shown by the three sub-collections, in which four species over the 21 detected accounted for the 78% of the whole collection, suggests that the cultivable biphenyl-utilizing bacteria likely were mainly selected by the soil characteristics occurring at the SIN Caffaro site rather than the plant species. The detection of *bphA* gene in 72% of the isolates indicates that these strains harbor the genetic information to initiate the upper pathway of PCB degradation. Moreover, the large majority of the isolates belonged to the genera *Arthrobacter, Microbacterium*, and *Pseudomonas*, taxa previously isolated from the rhizosphere of plants growing in PCB contaminated soils and known for their capacity to grow on biphenyl and, for some strains, to degrade PCB (Gilbert and Crowley, 1997; Leigh et al., 2007; Uhlik et al., 2011; Kurzawova et al., 2012). Most of the *bphA* sequences amplified from the rhizobacteria collection showed higher similarity with the biphenyl dioxygenase α subunit of *Pseudomonas pseudoalcaligenes* KF707, a model strain studied for its ability to metabolize PCBs through 2,3-dioxidation (Furukawa, 1994). This large group of sequences clusters together with the *bphA* sequence of the reference strain *Paraburkholderia xenovorans* LB400. The functional diversity between *Paraburkholderia xenovorans* LB400 and *Pseudomonas pseudoalcaligenes* KF707 is determined by minor differences in the gene sequence (Furukawa and Fujihara, 2008), whose detection was not possible in our analysis due to the short sequence coverage of the used primer set. Functional redundancy for PCB degradation in the SIN Caffaro soil is supported by *bph*A sequences displaying sequence divergence. A second cluster of sequences was affiliated with a gene previously sequenced in *Rhodococcus* spp., including the reference strain *R. jostii* RHA1, which shows a different range of substrates and a low degree of homology compared with the *bphA* proteins produced by *Pseudomonas* KF707 and *Paraburkholderia* LB400 (Masai et al., 1995). The *bphA* sequences of the SIN Caffaro isolates were not related to the phylogenetic identification of the isolates, reflecting that *bph* genes are frequently associated to mobile elements and can be spread through horizontal gene transfer (Pieper and Seeger, 2005). Catechol 2,3-dioxygenase activity was widespread throughout the collection and all the isolated strains harboring *bphA* gene also presented this activity. Biphenyl dioxygenase and catechol 2,3-dioxygenase are enzymes involved in the metabolism of several root-derived and xenobiotic aromatic compounds, so these results support the hypothesis that plants foster organic contaminant degradation by their root-associated bacteria (Fuchs et al., 2011). In particular, since catechol is a toxic metabolite produced by the degradation of biphenyl in the benzoate lower pathway, its degradation capability is essential for a complete mineralization of PCBs that can occur through co-metabolism by bacteria harboring the upper and/or lower degradation pathways (Leewis et al., 2016b).

Plant growth promotion activities have been frequently reported in bacteria from polluted soils (Croes et al., 2013; Thijs et al., 2014; Franchi et al., 2016), according to the ability of plants to select beneficial bacteria when growing under phytotoxic and stress conditions. Likewise, IAA production and ACC deaminase activity are PGP traits well represented in the cultivable biphenyl-utilizing rhizobacteria hosted by all the three plant species. Bacterial IAA production can influence root proliferation and elongation, thereby affecting nutrient and water uptake by plants (Lambrecht et al., 2000) besides phytoextraction and phytostabilization in soils contaminated by heavy metals (Ma et al., 2011). Moreover, indole and its derivatives are considered inter-kingdom signal molecules and play a role as biofilm regulators, a further feature in PGP bacteria-plant interactions (Lee et al., 2015). ACC deaminase activity is known to lower ACC level in plant cells interfering with ethylene biosynthesis and thereby decreasing plant stress response potentially deriving from chemical phytotoxicity (Glick, 2010). Therefore, the isolated strains showed a notable potential in supporting plant adaptation and growth in the highly polluted soil of the SIN Caffaro. Moreover, a significant fraction of isolates tolerated moderate saline, osmotic and temperature stresses. These phenotypes are not directly related to PGP or biodegradation activity, but could confer to the strains a higher fitness in soils with complex and uneven pollutant fingerprints and subjected to seasonal changes (Di Guardo et al., 2017).

Two strains belonging to the genus *Arthrobacter*, one of the most abundant in our collection, also proved to promote the growth of a model plant (tomato) under greenhouse conditions. Since *in vitro* screening alone is not always sufficient to evaluate the actual PGP potential of bacterial strains (Cardinale et al., 2015), this result is encouraging for further *in vivo* tests with plant species selected for rhizoremediation trials, considering that these bacteria are also well adapted to the heavy pollution of the SIN Caffaro soil.

## Conclusion

In this work we reported that three spontaneous plant species selected in the strongly PCB-polluted soil of a historical contaminated site, differentially affected the composition of the bacterial community in the root-associated soil fractions. The selective pressure imposed by the persistent chlorinated organic pollutants, heavy metals and metalloids in the soil, putatively affected the soil shared microbiome and determined an intrinsic functional potential for natural attenuation in the root-associated soil fractions. Rhizosphere bacterial strains harboring *bphA* gene and displaying catechol dioxygenase and PGP abilities, such as IAA production and ACC deaminase activity, are a potential resource for the improvement of plant growth and the detoxification in the heavily contaminated soils of SIN Caffaro, possibly exploitable for future rhizoremediation interventions.

## Author Contributions

Conceived and designed the experiments: LV, FM, and SB. Selected the sampling site and collected the samples: RM, SA, AD, and SB. Performed the experiments: LV, RM, FM, EC, and MF. Analyzed the data: LV, RM, MF, and FM. Contributed reagents/materials/analysis tools: DD and SB. Wrote the paper: LV, FM, RM, SB, and DD. All authors critically revised the manuscript.

## Conflict of Interest Statement

The authors declare that the research was conducted in the absence of any commercial or financial relationships that could be construed as a potential conflict of interest.

## Footnotes

1 https://code.google.com/p/ea-utils/wiki/FastqJoin

2 http://microbiomeutil.sourceforge.net/

3 www.ncbi.nlm.nih.gov

## References

[B1] Abhilash P. C. Dubey R. K. Tripathi V. Gupta V. K. Singh H. B. (2016). Plant growth-promoting microorganisms for environmental sustainability. *Trends Biotechnol.* 34 847–850. 10.1016/j.tibtech.2016.05.005 27265889

[B2] Abraham W.-R. Nogales B. Golyshin P. N. Pieper D. H. Timmis K. N. (2002). Polychlorinated biphenyl-degrading microbial communities in soils and sediments. *Curr. Opin. Microbiol.* 5 246–253. 10.1016/S1369-5274(02)00323-5 12057677

[B3] Albuquerque L. Franc L. Rainey F. A. Schumannc P. Nobred M. F. da Costa M. S. (2011). *Gaiella occulta* gen. nov., sp. nov., a novel representative of a deep branching phylogenetic lineage within the class *Actinobacteria* and proposal of *Gaiellaceae* fam. nov. and *Gaiellales* ord. nov. *Syst. Appl. Microbiol.* 34 595–599. 10.1016/j.syapm.2011.07.001 21899973

[B4] Anderson M. M. J. Gorley R. N. Clarke K. R. Clarke R. K. (2008). *PERMANOVA + for PRIMER: Guide to Software and Statistical Methods*. Plymouth: PRIMER-E.

[B5] Armiraglio S. Caccianiga M. Micheli E. Capretti A. (2009). Analisi preliminari sulla dinamica della vegetazione nel SIN Brescia-Caffaro. *Ann. Mus. Civ. Sc. Nat. Brescia* 2009 263–267.

[B6] Belimov A. A. Hontzeas N. Safronova V. I. Demchinskaya S. V. Piluzza G. Bullitta S. (2005). Cadmium-tolerant plant growth-promoting bacteria associated with the roots of Indian mustard (*Brassica juncea* L. Czern.). *Soil Biol. Biochem.* 37 241–250. 10.1016/j.soilbio.2004.07.033

[B7] Bell T. H. Hassan S. E.-D. Lauron-Moreau A. Al-Otaibi F. Hijri M. Yergeau E. (2014). Linkage between bacterial and fungal rhizosphere communities in hydrocarbon-contaminated soils is related to plant phylogeny. *ISME J.* 8 331–343. 10.1038/ismej.2013.149 23985744 PMC3906811

[B8] Berg G. Smalla K. (2009). Plant species and soil type cooperatively shape the structure and function of microbial communities in the rhizosphere. *FEMS Microbiol. Ecol.* 68 1–13. 10.1111/j.1574-6941.2009.00654.x 19243436

[B9] Bulgarelli D. Schlaeppi K. Spaepen S. Ver E. van Themaat L. Schulze-Lefert P. (2013). Structure and functions of the bacterial microbiota of plants. *Annu. Rev. Plant Biol.* 64 807–838. 10.1146/annurev-arplant-050312-120106 23373698

[B10] Caporaso J. G. Bittinger K. Bushman F. D. Todd Z. DeSantis T. Andersen G. L. (2010a). PyNAST: a flexible tool for aligning sequences to a template alignment. *Bioinformatics* 26 266–267. 10.1093/bioinformatics/btp636 19914921 PMC2804299

[B11] Caporaso J. G. Kuczynski J. Stombaugh J. Bittinger K. Bushman F. D. Costello E. K. (2010b). QIIME allows analysis of high–throughput community sequencing data. *Nat. Methods* 7 335–336. 10.1038/nmeth.f.303 20383131 PMC3156573

[B12] Cardinale M. Ratering S. Suarez C. Zapata Montoya A. M. Geissler-Plaum R. Schnell S. (2015). Paradox of plant growth promotion potential of rhizobacteria and their actual promotion effect on growth of barley (*Hordeum vulgare* L.) under salt stress. *Microbiol. Res.* 181 22–32. 10.1016/j.micres.2015.08.002 26640049

[B13] Cherif H. Marasco R. Rolli E. Ferjani R. Fusi M. Soussi A. (2015). Oasis desert farming selects environment-specific date palm root endophytic communities and cultivable bacteria that promote resistance to drought. *Environ. Microbiol. Rep.* 7 668–678. 10.1111/1758-2229.12304 26033617

[B14] Chouaia B. Rossi P. Montagna M. Ricci I. Crotti E. Damiani C. (2010). Molecular evidence for multiple infections as revealed by typing of *Asaia* bacterial symbionts of four mosquito species. *Appl. Environ. Microbiol.* 76 7444–7450. 10.1128/AEM.01747-10 20851960 PMC2976182

[B15] Croes S. Weyens N. Janssen J. Vercampt H. Colpaert J. V. Carleer R. (2013). Bacterial communities associated with *Brassica napus* L. grown on trace element-contaminated and non-contaminated fields: a genotypic and phenotypic comparison. *Microb. Biotechnol.* 6 371–384. 10.1111/1751-7915.12057 23594409 PMC3917472

[B16] Di Gregorio S. Azaizeh H. Lorenzi R. (2013). Biostimulation of the autochthonous microbial community for the depletion of polychlorinated biphenyls (PCBs) in contaminated sediments. *Environ. Sci. Pollut. Res.* 20 3989–3999. 10.1007/s11356-012-1350-x 23208754

[B17] Di Guardo A. Terzaghi E. Raspa G. Borin S. Mapelli F. Chouaia B. (2017). Differentiating current and past PCB and PCDD/F sources: the role of a large contaminated soil site in an industrialized city area. *Environ. Pollut.* 223 367–375. 10.1016/j.envpol.2017.01.033 28118998

[B18] Edgar R. C. (2010). Search and clustering orders of magnitude faster than BLAST. *Bioinformatics* 26 2460–2461. 10.1093/bioinformatics/btq461 20709691

[B19] Edgar R. C. (2013). UPARSE: highly accurate OUT sequences from microbial amplicon reads. *Nat. Methods* 10 996–998. 10.1038/nmeth.2604 23955772

[B20] Ferjani R. Marasco R. Rolli E. Cherif H. Cherif A. Gtari M. (2015). The date palm tree rhizosphere is a niche for plant growth promoting bacteria in the oasis ecosystem. *BioMed Res. Int.* 2015:153851 10.1155/2015/15385 PMC4383278 25866759

[B21] Franchi E. Agazzi G. Rolli E. Borin S. Marasco R. Chiaberge S. (2016). Exploiting hydrocarbon-degrading indigenous bacteria for bioremediation and phytoremediation of a multicontaminated soil. *Chem. Eng. Technol.* 39 1676–1684. 10.1002/ceat.201500573

[B22] Fuchs G. Boll M. Heider J. (2011). Microbial degradation of aromatic compounds — from one strategy to four. *Nat. Rev. Microbiol.* 9 803–816. 10.1038/nrmicro2652 21963803

[B23] Furukawa K. (1994). Molecular and evolutionary relationship of PCB-degrading bacteria. *Biodegradation* 5 289–300. 10.1007/BF00696466 7765839

[B24] Furukawa K. Fujihara H. (2008). Microbial degradation of polychlorinated biphenyls: biochemical and molecular features. *J. Biosci. Bioeng.* 105 443–449. 10.1263/jbb.105.433 18558332

[B25] Gilbert E. S. Crowley D. E. (1997). Plant compounds that induce polychlorinated biphenyl biodegradation by *Arthrobacter* sp. Strain B1B. *Appl. Environ. Microbiol.* 63 1933–1938. 9143124 10.1128/aem.63.5.1933-1938.1997 PMC168484

[B26] Glick B. R. (2010). Using soil bacteria to facilitate phytoremediation. *Biotechnol. Adv.* 28 367–374. 10.1016/j.biotechadv.2010.02.001 20149857

[B27] Haichar F. Z. Marol C. Berge O. Rangel-Castro J. I. Prosser J. I. Balesdent J. (2008). Plant host habitat and root exudates shape soil bacterial community structure. *ISME J.* 2 1221–1230. 10.1038/ismej.2008.80 18754043

[B28] Hammer Ø. Harper D. A. T. Ryan P. D. (2001). *PAST-Palaeontological Statistics, ver. 1.89*. Oslo: University of Oslo.

[B29] Hernández M. Dumont M. G. Yuan Q. Conrad R. (2015). Different bacterial populations associated with the roots and rhizosphere of rice incorporate plant-derived carbon. *Appl. Environ. Microbiol.* 81 2244–2253. 10.1128/AEM.03209-14 25616793 PMC4345361

[B30] Hu J. Qian M. Zhang Q. Cui J. Chunna Y. Su X. (2015). Sphingobium fuliginis HC3: a novel and robust isolated biphenyl and polychlorinated biphenyls-degrading bacterium without dead-end intermediates accumulation. *PLoS ONE* 10:e0122740 10.1371/journal.pone.0122740 PMC4395236 25875180

[B31] IARC (2015). *Polychlorinated and Polybrominated Biphenyls Volume 107 IARC Monographs on the Evaluation of Carcinogenic Risks to Humans. Lyon, France*. Available at: http://monographs.iarc.fr/ENG/Monographs/vol107/mono107.pdf PMC7681612 29905442

[B32] Ionescu M. Beranova K. Dudkova V. Kochankova L. Demnerova K. Macek T. (2009). Isolation and characterization of different plant associated bacteria and their potential to degrade polychlorinated biphenyls. *Int. Biodeterior. Biodegradation* 63 667–672. 10.1016/j.ibiod.2009.03.009

[B33] Iwai S. Chai B. Sul W. J. Cole J. R. Hashsham S. A. Tiedje J. M. (2010). Gene-targeted-metagenomics reveals extensive diversity of aromatic dioxygenase genes in the environment. *ISME J.* 4 279–285. 10.1038/ismej.2009.104 19776767 PMC2808446

[B34] Jayanna S. K. Gayathri D. (2015). Degradation of 2,4 dichlorobiphenyl via meta-cleavage pathway by *Pseudomonas* spp. consortium. *Curr. Microbiol.* 70 871–876. 10.1007/s00284-015-0800-3 25800378

[B35] Jugder B.-E. Ertan H. Lee M. Manefield M. Marquis C. P. (2015). Reductive dehalogenases come of age in biological destruction of organohalides. *Trends Biotechnol.* 33 595–610. 10.1016/j.tibtech.2015.07.004 26409778

[B36] Klindworth A. Pruesse E. Schweer T. Peplies J. Quast C. Horn M. (2013). Evaluation of general 16S ribosomal RNA gene PCR primers for classical and next-generation sequencing-based diversity studies. *Nucleic Acids Res.* 41:e1 10.1093/nar/gks808 PMC3592464 22933715

[B37] Kovach W. L. (1999). *MVSP–A Multivariate Statistical Package for Windows, ver. 3.1*. Pentraeth: Kovach Computing Services 137.

[B38] Kurzawova V. Stursa P. Uhlik O. Norkova K. Strohalm M. Lipov J. (2012). Plant–microorganism interactions in bioremediation of polychlorinated biphenyl-contaminated soil. *N. Biotechnol.* 30 15–22. 10.1016/j.nbt.2012.06.004 22728721

[B39] Lambrecht M. Okon Y. Van de Broek A. Vanderleyden J. (2000). Indole-3-acetic acid: a reciprocal signalling molecule in bacteria–plant interactions. *Trends Microbiol.* 8 298–300. 10.1016/S0966-842X(00)01732-7 10878760

[B40] Lee J.-H. Wood T. K. Lee J. (2015). Roles of indole as an interspecies and interkingdom signaling molecule. *Trends Microbiol.* 23 707–718. 10.1016/j.tim.2015.08.001 26439294

[B41] Leewis M. C. Uhlik O. Fraraccio S. McFarlin K. Kottara A. Glover C. (2016a). Differential impacts of willow and mineral fertilizer on bacterial communities and biodegradation in diesel fuel oil-contaminated soil. *Front. Microbiol.* 7:837 10.3389/fmicb.2016.00837 PMC4889597 27313574

[B42] Leewis M.-C. Uhlik O. Leigh M. B. (2016b). Synergistic processing of biphenyl and benzoate: carbon flow through the bacterial community in polychlorinated-biphenyl- contaminated soil. *Sci. Rep.* 6:22145 10.1038/srep22145 PMC4768254 26915282

[B43] Leigh M. B. Pellizari V. H. Uhlık O. Sutka R. Rodrigues J. Ostrom N. E. (2007). Biphenyl-utilizing bacteria and their functional genes in a pine root zone contaminated with polychlorinated biphenyls (PCBs). *ISME J.* 1 134–148. 10.1038/ismej.2007.26 18043623

[B44] Leigh M. B. Prouzová P. Macková M. Macek T. Nagle D. P. Fletcher J. S. (2006). Polychlorinated biphenyl (PCB)-degrading bacteria associated with trees in a PCB-contaminated site. *Appl. Environ. Microbiol.* 72 2331–2342. 10.1128/AEM.72.4.2331-2342.2006 16597927 PMC1449058

[B45] Letcher R. J. Bustnes J. O. Dietz R. Jenssen B. M. Jørgensen E. H. Sonne C. (2010). Exposure and effects assessment of persistent organohalogen contaminants in arctic wildlife and fish. *Sci. Total Environ.* 408 2995–3043. 10.1016/j.scitotenv.2009.10.038 19910021

[B46] Li Y. Liang F. Zhu Y. Wang F. (2013). Phytoremediation of a PCB-contaminated soil by alfalfa and tall fescue single and mixed plants cultivation. *J. Soils Sediments* 13 925–931. 10.1007/s11368-012-0618-6

[B47] Ma Y. Prasad M. N. V. Rajkumar M. Freitas H. (2011). Plant growth promoting rhizobacteria and endophytes accelerate phytoremediation of metalliferous soils. *Biotechnol. Adv.* 29 248–258. 10.1016/j.biotechadv.2010.12.001 21147211

[B48] Mapelli F. Marasco R. Rolli E. Barbato M. Cherif H. Guesmi A. (2013). Potential for plant growth promotion of rhizobacteria associated with *Salicornia* growing in Tunisian hypersaline soils. *Biomed Res. Int.* 2013:248078 10.1155/2013/248078 PMC3679824 23781499

[B49] Marasco R. Rolli E. Ettoumi B. Vigani G. Mapelli F. Borin S. (2012). A drought resistance-promoting microbiome is selected by root system under desert farming. *PLoS ONE* 7:e48479 10.1371/journal.pone.0048479 PMC3485337 23119032

[B50] Margesin R. Gander S. Zacke G. Gounot A. M. Schinner F. (2003). Hydrocarbon degradation and enzyme activities of cold-adapted bacteria and yeasts. *Extremophiles* 7 451–458. 10.1007/s00792-003-0347-2 12942349

[B51] Masai E. Yamada A. Healy J. M. Hatta T. Kimbara K. Fukuda M. (1995). Characterization of biphenyl catabolic genes of gram-positive polychlorinated biphenyl degrader *Rhodococcus* sp. strain RHA1. *Appl. Environ. Microbiol.* 6 2079–2085. 10.1128/aem.61.6.2079-2085.1995 PMC167480 7793929

[B52] McDonald D. Price M. N. Goodrich J. Nawrocki E. P. DeSantis T. Z. Probst A. (2012). An improved Greengenes taxonomy with explicit ranks for ecological and evolutionary analyses of bacteria and archaea. *ISME J.* 6 610–618. 10.1038/ismej.2011.139 22134646 PMC3280142

[B53] Pagé A. P. Yergeau E. Greer C. W. (2015). *Salix purpurea* stimulates the expression of specific bacterial xenobiotic degradation genes in a soil contaminated with hydrocarbons. *PLoS ONE* 10:7 10.1371/journal.pone.0132062 PMC4498887 26161539

[B54] Passatore L. Rossetti C. Juwarkard A. A. Massacci A. (2014). Phytoremediation and bioremediation of polychlorinated biphenyls (PCBs): state of knowledge and research perspectives. *J. Hazard. Mater.* 278 189–202. 10.1016/j.jhazmat.2014.05.051 24976127

[B55] Petric I. Bru D. Udikovic-Kolic N. Hrsaka D. Philippot L. Martin-Laurent F. (2011). Evidence for shifts in the structure and abundance of the microbial community in a long-term PCB-contaminated soil under bioremediation. *J. Hazard. Mater.* 195 254–260. 10.1016/j.jhazmat.2011.08.036 21885188

[B56] Pham T. T. Pino Rodriguez N. J. Hijri M. Sylvestre M. (2015). Optimizing polychlorinated biphenyl degradation by flavonoid-induced cells of the rhizobacterium *Rhodococcus erythropolis* U23A. *PLoS ONE* 10:e0126033 10.1371/journal.pone.0126033 PMC4430277 25970559

[B57] Pieper D. H. Seeger M. (2005). Bacterial metabolism of polychlorinated biphenyls. *J. Mol. Microbiol. Biotechnol.* 15 121–138. 10.1007/s00253-004-1810-4 18685266

[B58] Price M. N. Dehal P. S. Arkin A. P. (2010). FastTree 2 – approximately maximum-likelihood trees for large alignments. *PLoS ONE* 5:e9490 10.1371/journal.pone.0009490 PMC2835736 20224823

[B59] Quinete N. Schettgen T. Bertram J. Kraus T. (2014). Occurrence and distribution of PCB metabolites in blood and their potential health effects in humans: a review. *Environ. Sci. Pollut. Res.* 21 11951–11972. 10.1007/s11356-014-3136-9 24943885

[B60] Ridl J. Kolar M. Strejcek M. Strnad H. Stursa P. Paces J. (2016). Plants rather than mineral fertilization shape microbial community structure and functional potential in legacy contaminated soil. *Front. Microbiol.* 7:995 10.3389/fmicb.2016.00995 PMC4919359 27446035

[B61] Rolli E. Marasco R. Vigani G. Ettoumi B. Mapelli F. Deangelis M. L. (2015). Improved plant resistance to drought is promoted by the root-associated microbiome as a water stress-dependent trait. *Environ. Microbiol.* 17 316–331. 10.1111/1462-2920.12439 24571749

[B62] Sinkkonen S. Paasivirta J. (2000). Degradation half-life times of PCDDs, PCDFs and PCBs for environmental fate modeling. *Chemosphere* 40 943–949. 10.1016/S0045-6535(99)00337-9 10739030

[B63] Sylvestre M. (2013). Prospects for using combined engineered bacterial enzymes and plant systems to rhizoremediate polychlorinated biphenyls. *Environ. Microbiol.* 15 907–915. 10.1111/1462-2920.12007 23106850

[B64] Tamura K. Peterson D. Peterson N. Stecher G. Nei M. Kumar S. (2011). MEGA5: molecular evolutionary genetics analysis using maximum likelihood, evolutionary distance, and maximum parsimony methods. *Mol. Biol. Evol.* 28 2731–2739. 10.1093/molbev/msr121 21546353 PMC3203626

[B65] Thijs S. Van Dillewijn P. Sillen W. Truyens S. Holtappels M. D’Haen J. (2014). Exploring the rhizospheric and endophytic bacterial communities of *Acer pseudoplatanus* growing on a TNT-contaminated soil: towards the development of a rhizocompetent TNT-detoxifying plant growth promoting consortium. *Plant Soil* 385 15–36. 10.1007/s11104-014-2260-0

[B66] Touceda-Gonzalez M. Brader G. Antonielli L. Ravindran V. B. Waldner G. Friesl-Hanl W. (2015). Combined amendment of immobilizers and the plant growth-promoting strain *Burkholderia phytofirmans* PsJN favours plant growth and reduces heavy metal uptake. *Soil Biol. Biochem.* 91 140–150. 10.1016/j.soilbio.2015.08.038

[B67] Toussaint J.-P. Pham T. T. Barriault D. Sylvestre M. (2012). Plant exudates promote PCB degradation by a rhodococcal rhizobacteria. *Appl. Microbiol. Biotechnol.* 95 1589–1603. 10.1007/s00253-011-3824-z 22202970

[B68] Tu C. Teng Y. Luo Y. Sun X. Deng S. Li Z. (2011). PCB removal, soil enzyme activities, and microbial community structures during the phytoremediation by alfalfa in field soils. *J. Soils Sediments* 11 649–656. 10.1007/s11368-011-0344-5

[B69] Turrio-Baldassarri L. Abate V. Alivernini S. Battistelli C. L. Carasi S. Casella M. (2007). A study on PCB, PCDD/PCDF industrial contamination in a mixed urban-agricultural area significantly affecting the food chain and the human exposure. Part I: soil and feed. *Chemosphere* 67 1822–1830. 10.1016/j.chemosphere.2006.05.124 17234238

[B70] Turrio-Baldassarri L. Alivernini S. Carasi S. Casella M. Fuselli S. Iacovella N. (2009). PCB, PCDD and PCDF contamination of food of animal origin as the effect of soil pollution and the cause of human exposure in Brescia. *Chemosphere* 76 278–285. 10.1016/j.chemosphere.2009.03.002 19345979

[B71] Uhlik O. Strejcek M. Junkova P. Sanda M. Hroudova M. Vlcek C. (2011). Matrix-assisted laser desorption ionization (MALDI)–time of flight mass spectrometry- and MALDI biotyper-based identification of cultured biphenyl-metabolizing bacteria from contaminated horseradish rhizosphere soil. *Appl. Environ. Microbiol.* 77 6858–6866. 10.1128/AEM.05465-11 21821747 PMC3187117

[B72] Vergani L. Mapelli F. Zanardini E. Terzaghi E. Di Guardo A. Morosini C. (2017). Phyto-rhizoremediation of polychlorinated biphenyl contaminated soils: an outlook on plant-microbe beneficial interactions. *Sci. Total Environ.* 575 1395–1406. 10.1016/j.scitotenv.2016.09.218 27717569

[B73] Yergeau E. Sanschagrin S. Maynard C. St-Arnaud M. Greer C. W. (2014). Microbial expression profiles in the rhizosphere of willows depend on soil contamination. *ISME J.* 8 344–358. 10.1038/ismej.2013.163 24067257 PMC3906822

