# Supplementary material for: Bacteria Associated to Plants Naturally Selected in a Historical PCB Polluted Soil Show Potential to Sustain Natural Attenuation
Source: Front Microbiol. 2017 Jul 25;8:1385. doi: 10.3389/fmicb.2017.01385 (PMC5524726; doi:10.3389/fmicb.2017.01385)
Supplement: Supplementary file 1 [file Table_1.DOCX]

**SUPPLEMENTARY MATERIAL TO:**

**Bacteria associated to plants naturally selected in a historical PCB polluted soil show potential to sustain natural attenuation**

Lorenzo Vergani^1^, Francesca Mapelli^1^, Ramona Marasco^2^, Elena Crotti^1^, Marco Fusi^2^, Antonio Di Guardo^3^, Stefano Armiraglio^4^, Daniele Daffonchio^2,1^ and Sara Borin^1*^

^1^*Department of Food, Environmental and Nutritional Sciences, University of Milan, Milan, Italy,*

*^2^King Abdullah University of Science and Technology, Biological and Environmental Sciences and Engineering Division, Thuwal, Saudi Arabia*

*^3^Department of Science and High Technology, University of Insubria, Como, Italy*

*^4^Municipality of Brescia, Museum of Natural Sciences, Brescia, Italy*

*Correspondence:

Sara Borin

sara.borin@unimi.it

**Running title:** Plant-bacteria interactions support bioremediation potential

**SUPPLEMENTARY TABLES**

**Supplementary table 1.** The Good’s coverage value has been calculated for each sample in order to evaluate the quality of the sequencing process.

| **Bulk soil**  **(B)** | **Good’s coverage** |  | **Rhizosphere (R)** | **Good’s coverage** |  | **Soil Surrounding root (S)** | **Good’s coverage** |
| --- | --- | --- | --- | --- | --- | --- | --- |
| B1 | 0.98544 |  | CN1 | 0.99482 |  | CN1 | 0.987667 |
| B2 | 0.982849 |  | CN2 | 0.995275 |  | CN2 | 0.987637 |
| B3 | 0.929602 |  | CN3 | 0.997801 |  | CN3 | 0.986612 |
|  |  |  | DG1 | 0.990533 |  | DG1 | 0.985785 |
|  |  |  | DG2 | 0.991123 |  | DG2 | 0.990792 |
|  |  |  | DG3 | 0.989995 |  | DG3 | 0.986448 |
|  |  |  | MS1 | 0.998011 |  | MS1 | 0.904878 |
|  |  |  | MS2 | 0.999444 |  | MS2 | 0.986783 |
|  |  |  | MS3 | 0.984487 |  | MS3 | 0.986987 |

**Supplementary table 2. PERMANOVA for the 16S rRNA gene-based Illumina dataset**. **(A)** Main test comparison of the distance matrix generated according to OTUs distribution of bacterial communities associated to the three fractions (Rhizosphere, Soil surrounding root and Bulk) using one-way PERMANOVA. **(B)** Main test comparison of the distance matrix generated according to OTUs distribution of bacterial communities in the two fractions (Rhizosphere and Soil surrounding root) and three plant species (MS, NC and DG) using two-way PERMANOVA. Df= degree of freedom, MS = mean of square, F= F-statistic and *p* is the statistical *p* value. In bold the statistical significant terms.

| **A) Factor** | **Df** | **MS** | **F** | ***p*** |
| --- | --- | --- | --- | --- |
| **Fraction** | **2** | **2294.7** | **3.481** | **0.0007** |
| Residual | 18 | 659.21 |  |  |
| Total | 20 |  |  |  |

| **B) Factor** | **Df** | **MS** | **F** | **p** |
| --- | --- | --- | --- | --- |
| **Fraction** | **1** | **2320.4** | **4.7151** | **0.0024** |
| **Plant** | **2** | **1579.2** | **3.2089** | **0.0078** |
| FractionXPlant | 2 | 817.77 | 1.6617 | 0.0967 |
| Residual | 12 | 492.12 |  |  |

**Supplementary Table 3.** Estimates of components of variation of the two-way PERMANOVA between plant species and soil fractions with their interaction. In bold the statistical significant terms (see Supplementary Table 2).

| **Source** | **Estimate** | **Sq.root** | **%** |
| --- | --- | --- | --- |
| **Fraction** | **203.14** | **14.253** | **21** |
| **Plant** | **181.18** | **13.46** | **18** |
| FractionxPlant | 108.55 | 10.419 | 11 |
| Residual | 492.12 | 22.184 | 50 |

**Supplementary Table 4.** Shared OTUs of **(A)** soil fractions (R, S and B) and plant species (MS, NC and DG) in **(B)** rhizosphere and **(C)** soil surrounding root fractions.

| **A) Soil fractions** | **Number of elements** |
| --- | --- |
| B | 3022 |
| R | 3436 |
| S | 3436 |
| Overall number of unique elements | 3587 |
| Shared OTUs | 2831 |

| **B) Rhizosphere** | **Number of elements** |
| --- | --- |
| CN | 2854 |
| DG | 3146 |
| MS | 2326 |
| Overall number of unique elements | 3436 |
| Shared OTUs | 1922 |

| **C) Root surrounding root** | **Number of elements** |
| --- | --- |
| CN | 3117 |
| DG | 3072 |
| MS | 2930 |
| Overall number of unique elements | 3436 |
| Shared OTUs | 2558 |

**Supplementary Table 5. Relative abundance of the bacterial taxonomic groups in the soil samples associated to the plant species at the SIN Caffaro site.** CN = *Centaurea nigrescens* DG = *Dactylis glomerata,* MS = *Medicago sativa*. OTU_97_ are reported in percentage. Detail on the other taxonomic levels are provided in the Supplementary Table 6 as Excel file (Supplementary Table 6.xlxs).

| **(A) Phylum/Class** | **Rhizosphere (R)** | | | | | | | | |
| --- | --- | --- | --- | --- | --- | --- | --- | --- | --- |
|  | **CN1** | **CN2** | **CN3** | **DG1** | **DG2** | **DG3** | **MS1** | **MS2** | **MS3** |
| Acidobacteria | 2.9 | 4.0 | 4.2 | 10.6 | 7.7 | 8.0 | 0.17 | 2.7 | 1.6 |
| Actinobacteria | 55.7 | 44.0 | 50.2 | 21.8 | 36.0 | 35.0 | 5.42 | 31.6 | 23.4 |
| Alphaproteobacteria | 4.8 | 7.0 | 4.9 | 23.4 | 13.5 | 14.9 | 2.46 | 10.9 | 7.6 |
| Bacteroidetes | 1.2 | 2.7 | 0.9 | 8.5 | 5.8 | 7.8 | 0.41 | 4.9 | 1.3 |
| Betaproteobacteria | 4.2 | 3.6 | 3.4 | 5.5 | 5.1 | 3.4 | 0.73 | 4.3 | 2.8 |
| Chloroflexi | 13.6 | 11.3 | 12.8 | 6.4 | 9.5 | 8.2 | 0.85 | 5.3 | 4.9 |
| Deltaproteobacteria | 2.7 | 3.7 | 3.7 | 4.1 | 4.7 | 3.5 | 0.24 | 2.2 | 1.3 |
| Firmicutes | 5.0 | 14.8 | 7.7 | 1.1 | 4.4 | 5.0 | 1.33 | 11.2 | 10.3 |
| Gammaproteobacteria | 2.1 | 2.8 | 4.6 | 7.2 | 3.5 | 5.7 | 87.80 | 22.9 | 44.6 |
| Gemmatimonadetes | 1.4 | 0.8 | 1.1 | 1.1 | 1.2 | 0.9 | 0.01 | 0.4 | 0.2 |
| Other (<1%) | 1.0 | 1.3 | 1.7 | 2.5 | 2.3 | 1.8 | 0.04 | 0.7 | 0.3 |
| Planctomycetes | 0.8 | 0.8 | 1.1 | 2.7 | 1.7 | 1.8 | 0.03 | 0.5 | 0.3 |
| TM7 | 1.9 | 1.6 | 1.6 | 1.2 | 2.1 | 1.8 | 0.43 | 1.4 | 0.8 |
| Verrucomicrobia | 1.3 | 1.2 | 1.5 | 3.5 | 2.2 | 2.0 | 0.07 | 0.9 | 0.4 |
| WS3 | 1.5 | 0.4 | 0.6 | 0.5 | 0.4 | 0.4 | 0.01 | 0.1 | 0.2 |
|  |  |  |  |  |  |  |  |  |  |
|  |  |  |  |  |  |  |  |  |  |
| **(B) Phylum/Class** | **Soil Surrounding root (S)** | | | | | | | | |
|  | **CN1** | **CN2** | **CN3** | **DG1** | **DG2** | **DG3** | **MS1** | **MS2** | **MS3** |
| Acidobacteria | 14.6 | 12.0 | 11.3 | 10.3 | 14.9 | 11.0 | 23.5 | 15.2 | 12.8 |
| Actinobacteria | 23.8 | 25.7 | 31.6 | 39.8 | 31.3 | 40.2 | 15.2 | 23.5 | 30.2 |
| Alphaproteobacteria | 10.2 | 11.2 | 11.5 | 12.2 | 12.1 | 11.3 | 12.9 | 9.7 | 9.7 |
| Bacteroidetes | 4.4 | 4.1 | 3.8 | 3.2 | 3.6 | 3.5 | 6.4 | 3.0 | 2.5 |
| Betaproteobacteria | 6.6 | 7.8 | 5.9 | 5.2 | 6.1 | 4.8 | 4.6 | 7.9 | 7.0 |
| Chloroflexi | 11.1 | 11.3 | 12.2 | 8.8 | 8.1 | 8.8 | 5.5 | 10.3 | 11.1 |
| Deltaproteobacteria | 7.4 | 6.1 | 6.0 | 5.4 | 5.9 | 5.2 | 6.4 | 6.9 | 6.4 |
| Firmicutes | 1.4 | 1.3 | 1.5 | 0.9 | 0.7 | 0.8 | 1.1 | 1.2 | 1.4 |
| Gammaproteobacteria | 2.9 | 2.7 | 2.5 | 2.1 | 2.5 | 2.6 | 3.1 | 2.8 | 2.3 |
| Gemmatimonadetes | 3.0 | 3.4 | 2.5 | 1.6 | 1.8 | 1.3 | 2.7 | 4.0 | 3.5 |
| Other (<1%) | 2.7 | 2.7 | 2.1 | 2.3 | 2.7 | 2.2 | 4.4 | 2.7 | 2.3 |
| Planctomycetes | 4.4 | 4.1 | 3.2 | 3.0 | 3.9 | 3.0 | 4.6 | 4.1 | 3.4 |
| TM7 | 1.2 | 1.2 | 1.0 | 1.5 | 1.5 | 1.3 | 2.0 | 1.6 | 1.2 |
| Verrucomicrobia | 4.1 | 3.9 | 3.1 | 2.9 | 4.0 | 3.4 | 5.6 | 4.5 | 3.7 |
| WS3 | 2.1 | 2.6 | 1.9 | 0.8 | 0.9 | 0.6 | 1.9 | 2.6 | 2.4 |
|  |  |  |  |  |  |  |  |  |  |
|  |  |  |  |  |  |  |  |  |  |
|  |  |  |  |  |  |  |  |  |  |
|  |  |  |  |  |  |  |  |  |  |
| **(C) Phylum/Class** | **Bulk** | | |  |  |  |  |  |  |
|  | **B1** | **B2** | **B3** |  |  |  |  |  |  |
| Other (<1%) | 3.0 | 3.7 | 3.0 |  |  |  |  |  |  |
| WS3 | 3.2 | 2.7 | 2.0 |  |  |  |  |  |  |
| Verrucomicrobia | 4.9 | 5.5 | 4.8 |  |  |  |  |  |  |
| TM7 | 0.6 | 0.6 | 0.8 |  |  |  |  |  |  |
| Planctomycetes | 3.2 | 3.2 | 3.3 |  |  |  |  |  |  |
| Gemmatimonadetes | 4.5 | 3.7 | 3.6 |  |  |  |  |  |  |
| Gammaproteobacteria | 6.7 | 6.7 | 7.2 |  |  |  |  |  |  |
| Firmicutes | 1.7 | 0.8 | 0.8 |  |  |  |  |  |  |
| Deltaproteobacteria | 6.1 | 6.5 | 5.7 |  |  |  |  |  |  |
| Chloroflexi | 4.5 | 4.4 | 4.3 |  |  |  |  |  |  |
| Betaproteobacteria | 11.1 | 9.9 | 11.0 |  |  |  |  |  |  |
| Bacteroidetes | 12.1 | 11.6 | 14.0 |  |  |  |  |  |  |
| Alphaproteobacteria | 11.0 | 11.4 | 13.4 |  |  |  |  |  |  |
| Actinobacteria | 6.2 | 6.4 | 7.0 |  |  |  |  |  |  |
| Acidobacteria | 21.2 | 23.1 | 19.2 |  |  |  |  |  |  |

**Supplementary Table 6. Relative abundance of the bacterial taxonomic groups in the soil samples associated to the plant species at the SIN Caffaro site.** Relative abundance is indicated at the Order levels for each detected Phylum/Class. CN = *Centaurea nigrescens* DG = *Dactylis glomerata,* MS = *Medicago sativa*. OTU_97_ are reported in percentage.

The table is provided as Excel file.

**Supplementary Table 7.** Taxa distribution among the three plant species in **(A)** rhizosphere and **(B)** root surrounding soil fraction (left tables) and respective diversity indices (right tables).

| **(A) Phylum/Class** | **Rhizosphere** | | |  | **Diversity index** | **Rhizosphere** | | |
| --- | --- | --- | --- | --- | --- | --- | --- | --- |
|  | **CN** | **DG** | **MS** |  |  | **CN** | **DG** | **MS** |
| Acidobacteria | 3.7 | 8.7 | 1.5 |  | Dominance_D | 0.2835 | 0.1544 | 0.3224 |
| Actinobacteria | 50.0 | 30.9 | 20.1 |  | Simpson_1-D | 0.7165 | 0.8456 | 0.6776 |
| Alphaproteobacteria | 5.6 | 17.2 | 7.0 |  | Shannon_H | 1.834 | 2.224 | 1.585 |
| Bacteroidetes | 1.6 | 7.4 | 2.2 |  | Evenness_e^H/S | 0.4173 | 0.6163 | 0.3254 |
| Betaproteobacteria | 3.7 | 4.7 | 2.6 |  | Brillouin | 1.639 | 2.01 | 1.424 |
| Chloroflexi | 12.6 | 8.0 | 3.7 |  | Menhinick | 1.5 | 1.499 | 1.5 |
| Deltaproteobacteria | 3.3 | 4.1 | 1.2 |  | Margalef | 3.04 | 3.039 | 3.04 |
| Firmicutes | 9.2 | 3.5 | 7.6 |  | Equitability_J | 0.6773 | 0.8213 | 0.5854 |
| Gammaproteobacteria | 3.2 | 5.5 | 51.8 |  | Fisher_alpha | 4.894 | 4.892 | 4.894 |
| Gemmatimonadetes | 1.1 | 1.1 | 0.2 |  | Berger-Parker | 0.5 | 0.3087 | 0.518 |
| Other (<1%) | 1.3 | 2.2 | 0.3 |  |  |  |  |  |
| Planctomycetes | 0.9 | 2.1 | 0.3 |  |  |  |  |  |
| TM7 | 1.7 | 1.7 | 0.9 |  |  |  |  |  |
| Verrucomicrobia | 1.3 | 2.6 | 0.5 |  |  |  |  |  |
| WS3 | 0.8 | 0.4 | 0.1 |  |  |  |  |  |
|  |  |  |  |  |  |  |  |  |
|  |  |  |  |  |  |  |  |  |
| **(B) Phylum/Class** | **Root surrounding Soil** | | |  | **Diversity index** | **Root surrounding Soil** | | |
|  | **CN** | **DG** | **MS** |  |  | **CN** | **DG** | **MS** |
| Acidobacteria | 12.7 | 12.1 | 17.2 |  | Dominance_D | 0.1308 | 0.1849 | 0.1199 |
| Actinobacteria | 27.0 | 37.1 | 23.0 |  | Simpson_1-D | 0.8692 | 0.8151 | 0.8801 |
| Alphaproteobacteria | 11.0 | 11.9 | 10.7 |  | Shannon_H | 2.333 | 2.126 | 2.38 |
| Bacteroidetes | 4.1 | 3.4 | 4.0 |  | Evenness_e^H/S | 0.6871 | 0.5586 | 0.7206 |
| Betaproteobacteria | 6.8 | 5.4 | 6.5 |  | Brillouin | 2.11 | 1.916 | 2.154 |
| Chloroflexi | 11.5 | 8.6 | 9.0 |  | Menhinick | 1.5 | 1.5 | 1.501 |
| Deltaproteobacteria | 6.5 | 5.5 | 6.6 |  | Margalef | 3.04 | 3.04 | 3.041 |
| Firmicutes | 1.4 | 0.8 | 1.2 |  | Equitability_J | 0.8614 | 0.7849 | 0.879 |
| Gammaproteobacteria | 2.7 | 2.4 | 2.7 |  | Fisher_alpha | 4.894 | 4.894 | 4.896 |
| Gemmatimonadetes | 2.9 | 1.6 | 3.4 |  | Berger-Parker | 0.27 | 0.371 | 0.2302 |
| Other (<1%) | 2.5 | 2.4 | 3.1 |  |  |  |  |  |
| Planctomycetes | 3.9 | 3.3 | 4.0 |  |  |  |  |  |
| TM7 | 1.1 | 1.4 | 1.6 |  |  |  |  |  |
| Verrucomicrobia | 3.7 | 3.4 | 4.6 |  |  |  |  |  |
| WS3 | 2.2 | 0.7 | 2.3 |  |  |  |  |  |

**Supplementary Table 8. Identification, PGP traits, abiotic stress tolerance and degradation potential of the rhizobacteria associated to *Medicago sativa*, *Centaurea nigrescens* and *Dactylis glomerata*.** The list includes the taxonomic classification of all strains and the results of the physiological tests and *bphA* PCR assay performed. ACCd = ACC-deaminase activity; IAA = auxin production; P. Sol. = inorganic phosphate solubilization; Sid. = siderophore production; Prot. = protease production; EPS = exopolysaccharides release; NH_3_=ammonia production; PEG = polyethylene glycol; C 2,3 D = 2,3-catechol dioxygenase activity; bphA = positive PCR amplification of the *bphA* gene.

The table is provided as Excel file.

**Supplementary Table 9. Classification of the *bphA* gene sequences.** Information on the *bphA* gene. ID % = % of identity; Acc n = accession number of the closest relative sequence in NCBI. Strain code indicate the plant of origin (1: MS, 2: CN, 3: DG).

| **Strain** | **Species** | **Closest *bphA* sequence** | **ID %** | **Acc. N.** |
| --- | --- | --- | --- | --- |
| 1_12B | *Pseudomonas moraviensis* | Rhodococcus wratislaviensis strain P13 biphenyl 2,3-dioxygenase alpha subunit (bphA1) gene | 99 | KP972446 |
| 1_19 | *Arthrobacter scleromae* | Pseudomonas pseudoalcaligenes KF707 = NBRC biphenyl dioxygenase alpha subunit | 99 | AP014862 |
| 1_25 | *Arthrobacter scleromae* | Pseudomonas pseudoalcaligenes KF707 = NBRC biphenyl dioxygenase alpha subunit | 99 | AP014862 |
| 1_28 | *Streptomyces erythrochromogenes* | Rhodococcus opacus partial bphA1 gene for biphenyl 2,3-dioxygenase alpha subunit | 100 | AJ544524 |
| 1_37 | *Arthrobacter phenanthrenivorans* | Rhodococcus opacus partial bphA1 gene for biphenyl 2,3-dioxygenase alpha subunit | 100 | AJ544524 |
| 1_41 | *Microbacterium natoriense* | Rhodococcus wratislaviensis strain P13 biphenyl 2,3-dioxygenase alpha subunit (bphA1) gene | 99 | KP972446 |
| 1_45 | *Pseudomonas baetica* | Rhodococcus opacus partial bphA1 gene for biphenyl 2,3-dioxygenase alpha subunit | 100 | AJ544524 |
| 1_51 | *Microbacterium natoriense* | Rhodococcus wratislaviensis strain P13 biphenyl 2,3-dioxygenase alpha subunit (bphA1) gene | 99 | KP972446 |
| 1_58 | *Microbacterium natoriense* | Pseudomonas pseudoalcaligenes KF707 = NBRC biphenyl dioxygenase alpha subunit | 99 | AP014862 |
| 2_10 | *Bacillus aryabhattai* | Pseudomonas pseudoalcaligenes KF707 = NBRC biphenyl dioxygenase alpha subunit | 99 | AP014862 |
| 2_13 | *Acinetobacter calcoaceticus* | Pseudomonas pseudoalcaligenes KF707 = NBRC biphenyl dioxygenase alpha subunit | 99 | AP014862 |
| 2_14B | *Agromyces terreus* | Pseudomonas pseudoalcaligenes KF707 = NBRC biphenyl dioxygenase alpha subunit | 99 | AP014862 |
| 2_15B | *Acinetobacter calcoaceticus* | Pseudomonas pseudoalcaligenes KF707 = NBRC biphenyl dioxygenase alpha subunit | 99 | AP014862 |
| 2_16 | *Rahnella aquatilis* | Pseudomonas pseudoalcaligenes KF707 = NBRC biphenyl dioxygenase alpha subunit | 99 | AP014862 |
| 2_17 | *Bacillus cereus* | Pseudomonas pseudoalcaligenes KF707 = NBRC biphenyl dioxygenase alpha subunit | 99 | AP014862 |
| 2_28 | *Microbacterium takaoensis* | Pseudomonas pseudoalcaligenes KF707 = NBRC biphenyl dioxygenase alpha subunit | 99 | AP014862 |
| 2_34 | *Microbacterium yannicii* | Pseudomonas pseudoalcaligenes KF707 = NBRC biphenyl dioxygenase alpha subunit | 99 | AP014862 |
| 2_36 | *Microbacterium yannicii* | Pseudomonas pseudoalcaligenes KF707 = NBRC biphenyl dioxygenase alpha subunit | 99 | AP014862 |
| 2_38A | *Agromyces cerinus* | Pseudomonas pseudoalcaligenes KF707 = NBRC biphenyl dioxygenase alpha subunit | 99 | AP014862 |
| 2_42 | *Microbacterium phyllosphaerae* | Pseudomonas pseudoalcaligenes KF707 = NBRC biphenyl dioxygenase alpha subunit | 99 | AP014862 |
| 2_43 | *Agromyces cerinus* | Pseudomonas pseudoalcaligenes KF707 = NBRC biphenyl dioxygenase alpha subunit | 99 | AP014862 |
| 2_46 | *Pseudomonas salomonii* | Pseudomonas pseudoalcaligenes KF707 = NBRC biphenyl dioxygenase alpha subunit | 99 | AP014862 |
| 2_47 | *Microbacterium arthrosphaerae* | Pseudomonas pseudoalcaligenes KF707 = NBRC biphenyl dioxygenase alpha subunit | 99 | AP014862 |
| 2_49 | *Pseudomonas koreensis* | Pseudomonas pseudoalcaligenes KF707 = NBRC biphenyl dioxygenase alpha subunit | 99 | AP014862 |
| 2_5 | *Bacillus megaterium* | Pseudomonas pseudoalcaligenes KF707 = NBRC biphenyl dioxygenase alpha subunit | 99 | AP014862 |
| 2_55 | *Promicromonospora sukumoe* | Pseudomonas pseudoalcaligenes KF707 = NBRC biphenyl dioxygenase alpha subunit | 99 | AP014862 |
| 2_57 | *Pseudomonas putida* | Rhodococcus opacus partial bphA1 gene for biphenyl 2,3-dioxygenase alpha subunit | 100 | AJ544524 |
| 2_9 | *Paenibacillus lautus* | Pseudomonas pseudoalcaligenes KF707 = NBRC biphenyl dioxygenase alpha subunit | 99 | AP014862 |

| **Strain** | **Species** | **Closest *bphA* sequence** | **ID %** | **Acc. N.** |
| --- | --- | --- | --- | --- |
| 3_1 | *Microbacterium yannicii* | Pseudomonas pseudoalcaligenes KF707 = NBRC biphenyl dioxygenase alpha subunit | 99 | AP014862 |
| 3_12 | *Microbacterium yannicii* | Pseudomonas pseudoalcaligenes KF707 = NBRC biphenyl dioxygenase alpha subunit | 99 | AP014862 |
| 3_13 | *Microbacterium yannicii* | Pseudomonas pseudoalcaligenes KF707 = NBRC biphenyl dioxygenase alpha subunit | 99 | AP014862 |
| 3_15A | *Acinetobacter calcoaceticus* | Pseudomonas pseudoalcaligenes KF707 = NBRC biphenyl dioxygenase alpha subunit | 99 | AP014862 |
| 3_19 | *Arthrobacter nitroguajacolicus* | Pseudomonas pseudoalcaligenes KF707 = NBRC biphenyl dioxygenase alpha subunit | 99 | AP014862 |
| 3_2 | *Acinetobacter calcoaceticus* | Rhodococcus opacus partial bphA1 gene for biphenyl 2,3-dioxygenase alpha subunit | 100 | AJ544524 |
| 3_21 | *Bacillus toyonensis* | Pseudomonas pseudoalcaligenes KF707 = NBRC biphenyl dioxygenase alpha subunit | 99 | AP014862 |
| 3_22 | *Arthrobacter oxydans* | Pseudomonas pseudoalcaligenes KF707 = NBRC biphenyl dioxygenase alpha subunit | 99 | AP014862 |
| 3_32A | *Pseudomonas putida* | Pseudomonas pseudoalcaligenes KF707 = NBRC biphenyl dioxygenase alpha subunit | 99 | AP014862 |
| 3_33 | *Arthrobacter oryzae* | Pseudomonas pseudoalcaligenes KF707 = NBRC biphenyl dioxygenase alpha subunit | 99 | AP014862 |
| 3_36 | *Microbacterium natoriense* | Rhodococcus wratislaviensis strain P13 biphenyl 2,3-dioxygenase alpha subunit | 99 | KP972446 |
| 3_38A | *Bacillus cereus* | Pseudomonas pseudoalcaligenes KF707 = NBRC biphenyl dioxygenase alpha subunit | 99 | AP014862 |
| 3_48 | *Arthrobacter scleromae* | Pseudomonas pseudoalcaligenes KF707 = NBRC biphenyl dioxygenase alpha subunit | 99 | AP014862 |
| 3_55 | *Pseudomonas putida* | Pseudomonas pseudoalcaligenes KF707 = NBRC biphenyl dioxygenase alpha subunit | 99 | AP014862 |
| 3_57 | *Arthrobacter humicola* | Pseudomonas pseudoalcaligenes KF707 = NBRC biphenyl dioxygenase alpha subunit | 99 | AP014862 |
| 3_6 | *Buttiauxella agrestis* | Pseudomonas pseudoalcaligenes KF707 = NBRC biphenyl dioxygenase alpha subunit | 99 | AP014862 |
| 3_9 | *Acinetobacter calcoaceticus* | Pseudomonas pseudoalcaligenes KF707 = NBRC biphenyl dioxygenase alpha subunit | 99 | AP014862 |

**Supplementary Table 10.** Identification, PGP activity, abiotic stress traits and bioremediation potential of the eleven strains selected for the *in vivo* PGP test

| **Plant** | **Strain** | **Identification^#^**  **(% identity)** | **PGP activity** | | | | | | |  | **Abiotic stress tolerance** | | | |  | **Bioremediation potential** | |
| --- | --- | --- | --- | --- | --- | --- | --- | --- | --- | --- | --- | --- | --- | --- | --- | --- | --- |
|  |  |  | **ACCd** | **IAA** | **P sol** | **Sid** | **Prot** | **EPS** | **NH_3_** |  | **4°C** | **42°C** | **20%PEG** | **5% NaCl** |  | **C 2,3 D** | ***bphA*** |
| **MS** | 1-45 | *Pseudomonas baetica* (98) | 0 | 1 | 0 | 0 | 1 | 0 | 0 |  | 1 | 0 | 1 | 0 |  | 1 | 1 |
|  | 1-8 | *Pseudomonas salomonii (99)* | 0 | 1 | 0 | 0 | 0 | 0 | 0 |  | 1 | 1 | 1 | 1 |  | 1 | 0 |
| **CN** | 2-15B | *Acinetobacter calcoaceticus* (99) | 0 | 0 | 1 | 0 | 0 | 0 | 1 |  | 1 | 1 | 1 | 1 |  | 1 | 1 |
|  | 2-2 | *Pseudomonas reinekei (99)* | 0 | 0 | 0 | 1 | 0 | 0 | 1 |  | 1 | 1 | 1 | 1 |  | 1 | 0 |
|  | 2-22B | *Pseudomonads baetica (99)* | 0 | 1 | 0 | 1 | 1 | 0 | 1 |  | 1 | 0 | 1 | 1 |  | 1 | 0 |
|  | 2-30 | *Arthrobacter aurescens (99)* | 0 | 1 | 0 | 0 | 0 | 0 | 0 |  | 1 | 1 | 1 | 1 |  | 1 | 0 |
|  | 2-50 | *Arthrobacter nitroguajacolicus* (99) | 1 | 0 | 0 | 0 | 1 | 0 | 0 |  | 1 | 0 | 1 | 1 |  | 1 | 1 |
| DG | 3-15A | *Acinetobacter calcoaceticus (99)* | 0 | 1 | 0 | 0 | 0 | 0 | 0 |  | 1 | 1 | 1 | 1 |  | 1 | 1 |
|  | 3-15B | *Arthrobacter aurescens (99)* | 1 | 1 | 0 | 1 | 1 | 0 | 1 |  | 1 | 0 | 1 | 1 |  | 0 | 0 |
|  | 3-24 | *Pseudomonas baetica (99)* | 0 | 1 | 0 | 0 | 1 | 0 | 1 |  | 1 | 0 | 1 | 0 |  | 1 | 1 |
|  | 3-59 | *Curtobacterium flaccumfaciens (99)* | 1 | 1 | 0 | 0 | 1 | 1 | 0 |  | 0 | 0 | 1 | 1 |  | 1 | 0 |

# Identification was determined according to the partial sequence of the 16S rRNA gene

ACCd: ACC-deaminase activity; IAA: auxin production; Sol P: inorganic phosphate solubilization; Sid: siderophore production; Prot. = protease production; ESP: exopolysaccharide production; NH_3_=ammonia production; PEG = polyethylene glycol; C 2,3 D = 2,3-catechol dioxygenase activity; *bphA* = positive PCR amplification of the *bphA* gene.

0/1: absence/presence of the activity.

**SUPPLEMENTARY FIGURES**

**Supplementary Figure 1.** **Identification of biphenyl-utilizing rhizobacteria from spontaneous plants.** Phylogenetic classification is reported at the genus level for the bacteria isolated from the rhizosphere of MS*,* CN and DG.


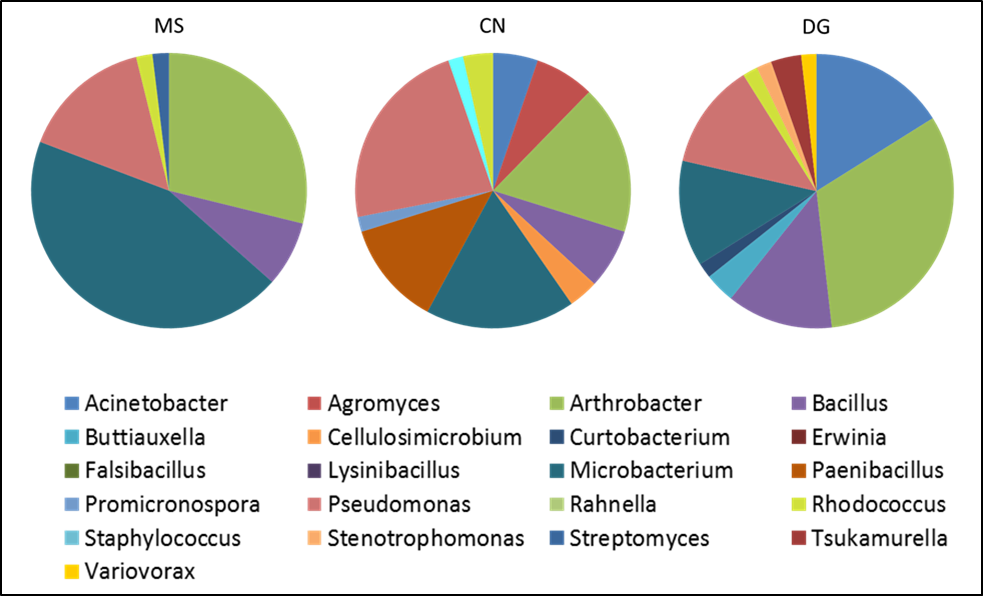


**Supplementary Figure 2. Phylogenetic tree of the *bphA* gene sequences of the isolated strains.** Reference strains *P. xenovorans* LB400 and *R. jostii* RHA1 are indicated in bold.

**
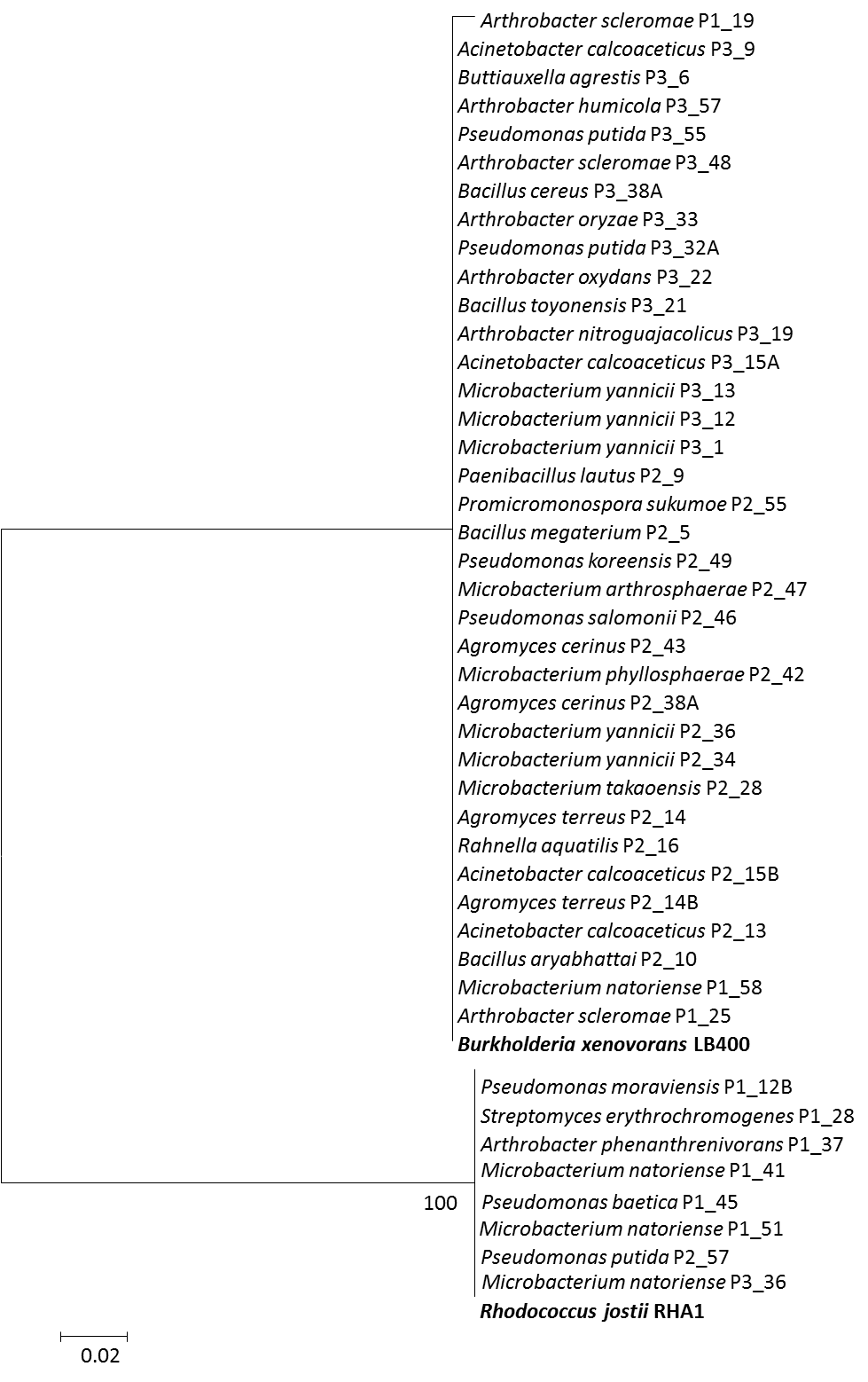
**

**Supplementary Figure 3. Selection of PGP bacteria for the *in vivo* assay.** Cluster analysis combining the PGP activities and the abiotic stress tolerance traits of the strains. Black dots and codes are indicated for the selected PGP strains.

**
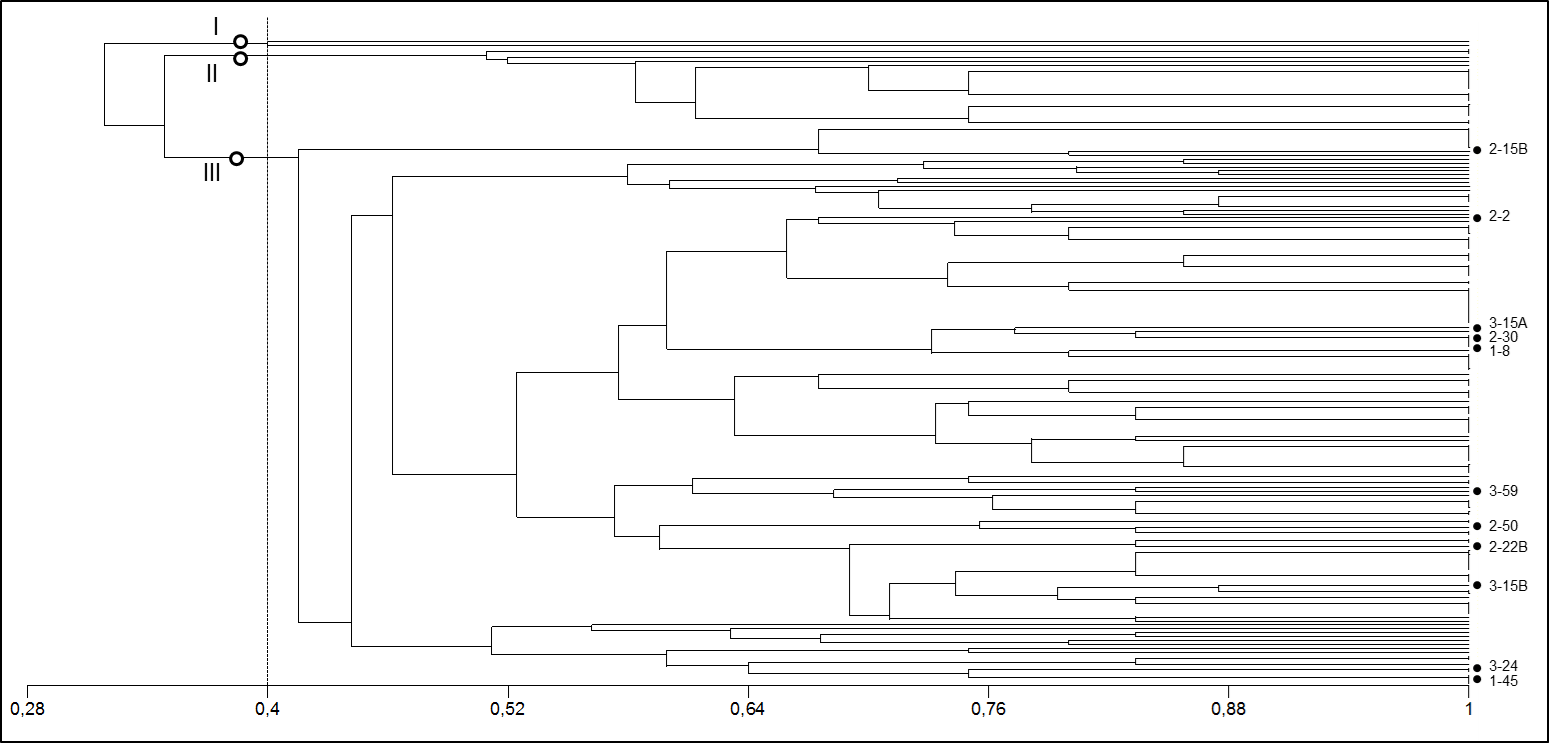
**
